# Genomic landscape of the immunogenicity regulation in skin melanomas with diverse tumor mutation burden

**DOI:** 10.3389/fimmu.2022.1006665

**Published:** 2022-10-28

**Authors:** George Georgoulias, Apostolos Zaravinos

**Affiliations:** ^1^ Department of Life Sciences, School of Sciences, European University Cyprus, Nicosia, Cyprus; ^2^ Cancer Genetics, Genomics and Systems Biology laboratory, Basic and Translational Cancer Research Center (BTCRC), Nicosia, Cyprus

**Keywords:** skin melanoma, tumor mutation burden (TMB), immune signatures, immune checkpoint inhibition therapy, patient response, tumor-infiltrating lymphocytes, tumor microenvironment

## Abstract

Skin melanoma cells are tightly interconnected with their tumor microenvironment (TME), which influences their initiation, progression, and sensitivity/resistance to therapeutic interventions. An immune-active TME favors patient response to immune checkpoint inhibition (ICI), but not all patients respond to therapy. Here, we assessed differential gene expression in primary and metastatic tumors from the TCGA-SKCM dataset, compared to normal skin samples from the GTEx project and validated key findings across 4 independent GEO datasets, as well as using immunohistochemistry in independent patient cohorts. We focused our attention on examining the expression of various immune receptors, immune-cell fractions, immune-related signatures and mutational signatures across cutaneous melanomas with diverse tumor mutation burdens (TMB). Globally, the expression of most immunoreceptors correlated with patient survival, but did not differ between TMB^high^ and TMB^low^ tumors. Melanomas were enriched in *“naive T-cell”, “effector memory T-cell”, “exhausted T-cell”, “resting Treg T-cell”* and *“Th1-like”* signatures, irrespective of their *BRAF*, *NF1* or *RAS* mutational status. Somatic mutations in *IDO1* and *HLA-DRA* were frequent and could be involved in hindering patient response to ICI therapies. We finally analyzed transcriptome profiles of ICI-treated patients and associated their response with high levels of IFNγ, Merck18, CD274, CD8, and low levels of myeloid-derived suppressor cells (MDSCs), cancer-associated fibroblasts (CAFs) and M2 macrophages, irrespective of their TMB status. Overall, our findings highlight the importance of pre-existing T-cell immunity in ICI therapeutic outcomes in skin melanoma and suggest that TMB^low^ patients could also benefit from such therapies.

## Introduction

Cutaneous melanomas are among the most immunogenic cancers (1), with an increasing incidence rate worldwide (2). They have an increased mutation rate as a result of exposure to UV radiation (2, 3) and are very heterogenous with different mutational subtypes, being mainly sorted according to the mutational status of *BRAF*, *NRAS* and *NF1* (4–6). Additionally, skin melanomas can be classified across five main immune subtypes; wound healing, IFN-γ dominant, inflammatory, lymphocyte depleted and TGF-β dominant; whereas very few of them are immunologically quiet (7).

The tumor microenvironment (TME) is the ecosystem surrounding a tumor and includes the extracellular matrix, blood vessels and stromal cells. It also encompasses a diverse number of immune cells, such as dendritic cells (DCs), neutrophils, natural killer (NK) cells, T-cells and B-cells, as well as immunosuppressors, including myeloid-derived suppressor cells (MDSCs), regulatory T (Treg) cells, tumor-associated macrophages (TAMs) or cancer-associated fibroblasts (CAFs). All these, constitute an ecosystem where they interact with the tumor cells bidirectionally, modulating the malignant phenotype (8). An immune-active TME has been shown to favor clinical response to immune checkpoint inhibition (ICI) therapies with anti-CTLA-4 and anti-PD-1 mAbs (9–11). The absence of tumor-infiltrating lymphocytes (TILs) in the TME on the other hand, predicts sentinel lymph node metastasis and survival (12). Combination immunotherapy or dual ICI (anti-PD-1 plus anti-CTLA-4) has recently shown impressive response rates in metastatic patients. However, half of them had significant toxicity from the treatment regimen (13, 14).

The tumor’s relationship with immune cells within the TME can remarkably influence cancer cell proliferation, progression, and metastasis (15). This unique immunogenicity renders skin melanoma as a paradigm for tumor-immune interactions and is driven by a high mutational burden (TMB), which can increase the tumor’s probability to generate immunogenic neoantigens, making it easier for the immune system to recognize cancer cells and elicit effective immune responses against them (16–18). Patients with high TMB are also likely to be more responsive to immunotherapy (19, 20). However, despite the promising therapeutic outcome that most ICI therapies provide to metastatic patients, most of them will not respond, exhibiting early (primary) or late (adaptive) resistance and relapse (21).

Here, we delved into the expression of a group of activating and inhibitory immune receptors in the TME of skin melanoma patients with diverse TMB. We also examined immune-related signatures, fractions of immune-cells and mutational signatures across tumors with a low or high TMB. Our results indicate that elevated expression levels of *TIGIT*, *IDO1* and *LAG3*, other than *PD-1, PD-L1/2* and *CTLA-4*, associate with the patients’ overall and disease-free survival, but not with the TMB, corroborating that immunogenicity in these tumors is affected by other factors as well. In addition, we found that skin melanomas are significantly enriched in the “naive T-cell”, “effector memory T-cell”, “exhausted T-cell”, “resting Treg T-cell” and “Th1-like” signatures, irrespective of their *BRAF*, *NF1* and *RAS* mutational status. We also show that despite the similar immune-cell fractions between TMB^high^ and TMB^low^ tumors, the first have a higher ratio of M1/M2 macrophages. Our data further support that somatic mutations in *IDO1* and *HLA-DRA* are frequent and could be involved in hindering patient response to ICI therapies. We finally provide evidence that TMB alone is not the best predictor of immunotherapy response and therefore, anti-PD-1/anti-CTLA-4 monotherapy or combination ICI therapy could also be applied to TMB^low^ patients.

## Materials and methods

### NGS data extraction and analysis

We extracted whole exome and RNA-seq data from the TCGA-SKCM dataset, containing 461 primary and metastatic skin melanoma samples, in total. All data, including patient clinicopathological information and MAF files, were assessed from GDC Data Portal (https://portal.gdc.cancer.gov/). Apart from one matched blood sample from the TCGA cohort that was used as control, we included normal skin samples from the GTEx project (https://gtexportal.org/) for differential gene expression analysis, totaling to 557 controls. TCGA and GTEx samples were re-aligned to the hg38 genome and were processed using a uniform bioinformatic pipeline, to eliminate batch effects.

Differential gene expression was identified between skin melanoma and matched TCGA normal and GTEx normal skin data, using limma with cut-off |log_2_FC>2| for upregulation and |log_2_FC<1| for downregulation, along with adjusted p<0.05. The B-statistic was used to sort the differentially expressed genes. We then performed Gene Ontology (GO) enrichment analysis for the top 250 up- and down-regulated genes in primary (or metastatic) skin melanomas, respectively, using Enrichment Analysis Visualization Appyter. Similar gene sets from GO analysis were clustered together using Uniform Manifold Approximation and Projection (UMAP) (22) and the significantly enriched (adjusted p<0.05) GO terms for biological processes (GO-BP), molecular function (GO-MF) and cellular component (GO-CC) were highlighted.

We focused on the expression of some well-known immune checkpoints, including *PD-1, PD-L1/2*, *CTLA-4, TIGIT*, *IDO1/2* and other prospective immunoreceptors (*LAG3, VTCN1*, *VISTA, ILT2* and *ILT4*). To calculate each gene’s expression, we used one-way ANOVA, and the disease state (skin melanoma or matched TCGA normal and GTEx normal skin samples) as variable to calculate differential expression. The expression data were first log_2_(TPM+1) transformed for differential analysis and the |log_2_FC| was defined as median (skin melanoma) - median (matched TCGA normal and GTEx samples), as explained before (23, 24).

### Validation of deregulated genes using independent GEO datasets

Four independent studies from the Gene Expression Omnibus (GEO) repository were analyzed for subsequent validation of the top deregulated genes in primary (or metastatic) melanomas against their adjacent normal skin samples, or between primary and metastatic melanomas, depending on the study. In specific, we obtained microarray data from the studies with the following GEO accession numbers: GSE8401, containing 31 primary and 52 metastatic melanomas (25, 26); GSE7553, 2 *in-situ* melanomas, 14 primary, 40 metastatic melanomas and 4 normal skin samples (27); GSE46517, 31 primary, 73 metastatic melanomas and 7 normal skin samples (28); and GSE15605, composed of 46 primary and 12 metastatic melanomas, as well as 16 normal skin samples. Data were analyzed using limma with vooma transformation in R (29). P-values were adjusted using Benjamini & Hochberg (FDR) and the significance threshold was set at p<0.05. The top 250 differentially expressed genes (ranked by p-value) were obtained either between primary and metastatic melanomas, or between each of those and their corresponding normal skin samples. UMAP, boxplots, and expression density plots were retrieved to assess normalization status and sample groupings. Volcano plots and mean difference (MD) plots were used to visualize differentially expressed genes. Adjusted p-value histograms were generated using hist to view the distribution of the p-values in the analysis results. Moderated t-statistic quantile-quantile (q-q) plots were used to check the variation in the data.

### Immune-related gene signatures

We compared immune-related gene signatures between cutaneous melanoma and control samples (matched TCGA and GTEx normal data), as well as between BRAF hotspot mutants (BRAF^mut^, n=147), NF1 mutants (NF1^mut^, n=27), RAS hotspot mutants (RAS^mut^, n=91) and triple-wild type (Triple^WT^, n=47) tumors, using GEPIA2 (30). The signatures were specific for naive T-cells (*CCR7, LEF1, TCF7* and *SELL*); effector T-cells (*CX3CR1, FGFBP2* and *FCGR3A*); effector memory T-cells (*PDCD1, DUSP4, GZMK, GZMA* and *IFNG*); central memory T-cells (*CCR7, SELL* and *IL7R*); resident memory T-cells (*CD69, ITGAE, CXCR6* and *MYADM*); exhausted T-cells (*HAVCR2, TIGIT, LAG3, PDCD1, CXCL13* and *LAYN*); resting Tregs (*FOXP3, IL2RA*); effector Tregs (*FOXP3, CTLA-4, CCR8* and *TNFRSF9*); and Th1-like cells (*CXCL13, HAVCR2, IFNG, CXCR3, BHLHE40* and *CD4*). The |log_2_FC>1| and p<0.01 (ANOVA) were used to assess differences with statistical significance between groups. Principal component analysis (PCA) was used to automatically perform dimensionality reduction on data from the TCGA-SKCM dataset and normal suprapubic skin (not exposed to the sun), based on the expression of these signatures in the samples. The expression of specific immune-checkpoints was also explored individually across the different molecular or immune subtypes (C1, wound healing; C2, IFN-gamma dominant; C3, inflammatory; C4, lymphocyte depleted; C5, immunologically quiet; C6, TGF-b dominant) (7).

### Commutation analysis and comparison of immunostimulators and immunoinhibitors between TMB^high^ and TMB^low^ tumors

We used iCoMut Beta 0.21 for FireBrowse to categorize skin melanomas into three TMB subgroups, based on the mutational distribution quartiles. The lower quartile contained tumors with a low mutation rate, i.e., <7.4 synonymous and non-synonymous (total) mutations/Mb or <5.14 non-synonymous mutations/MB (also termed as “TMB^low^”). The upper quartile involved tumors with an increased rate of mutation, i.e., >30 total mutations/Mb or >20 non-synonymous mutations/MB (“TMB^high^”). Among the TMB^high^ subgroup, 18 tumors with >81 total mutations/Mb were considered as “extremely hypermutated”. The rest 50% of samples was termed “TMB-intermediate” (TMB^int^, >7.42 & <30 total mut/Mb). Tumor stratification based on their TMB (synonymous and non-synonymous mutations) was also reflected on their neoantigen burden, being significantly higher among TMB^high^ tumors (68,263 neoantigens, 734.5 ± 695.7; median ± SD) versus TMB^int^ (34,473 neoantigens, 211 ± 151.2) and TMB^low^ tumors (4,929 neoantigens 57 ± 52.3). Maftools (31) was also used to compare oncoplots between TMB^high^ and TMB^low^ tumors.

The mutation rate was then correlated with the expression of either activating or inhibitory immune receptors within each TMB subgroup. In specific, we compared the expression of 49 immunostimulators (*BTNL2, C10orf54, CD27, CD274, CD276, CD28, CD40, CD40LG, CD48, CD70, CD80, CD86, CXCL12, CXCR4, ENTPD1, HHLA2, ICOS, ICOSLG, IL2RA, IL6, IL6R, KLRC1, KLRK1, LTA, MICA, MICB, NT5E, PDCD1LG2, PVR, RAET1E, TMEM173, TMIGD2, TNFRSF13B, TNFRSF13C, TNFRSF14, TNFRSF17, TNFRSF18, TNFRSF25, TNFRSF4, TNFRSF8, TNFRSF9, TNFSF13, TNFSF13B, TNFSF14, TNFSF15, TNFSF18, TNFSF4, TNFSF9 and ULBP1*) and 23 immunoinhibitors (*ADORA2A, BTLA, CD160, CD244, CD96, CSF1R, CTLA4, HAVCR2, IDO1, IL10, IL10RB, KDR, KIR2DL1, KIR2DL2, KIR2DL3, LAG3, LGALS9, PDCD1, PVRL2, TGFB1, TGFBR1, TIGIT and VTCN1*) across TMB^high^, TMB^int^ and TMB^low^ melanoma tumors.

### Mutational signatures and cancer driver genes

We extracted and analyzed single base substitutions (SBS) and doublet base substitutions (DBS) using SigProfiler’s MatrixGenerator and Extractor, as previously described in detail (32, 33). SBS signatures were identified using 96 different contexts, considering also the bases 5’ and 3’ from the mutated base. DBS signatures were generated after the concurrent modification of two consecutive nucleotide bases (34). The extracted mutational signatures were then compared against the ones found in COSMIC v3.2 (https://cancer.sanger.ac.uk/signatures/). Each signature’s contribution was calculated separately for primary and metastatic skin melanomas. Cancer driver mutations were identified using IntOGen (35).

### Cell-type fractions

We analyzed each tumor’s cell type fraction by extracting data from the Cancer Immunome Database (TCIA) (36). The absolute values and the quanTIseq computational pipeline were used to quantify tumoral immune contexture (37), focusing on B cells, M1/M2 macrophages, neutrophils, monocytes, NK cells, non-regulatory CD4+ and CD8+ T cells, regulatory CD4+ T cells (Tregs) and dendritic cells.

### Immunohistochemistry and evaluation of TIL load

**Figure 1:**
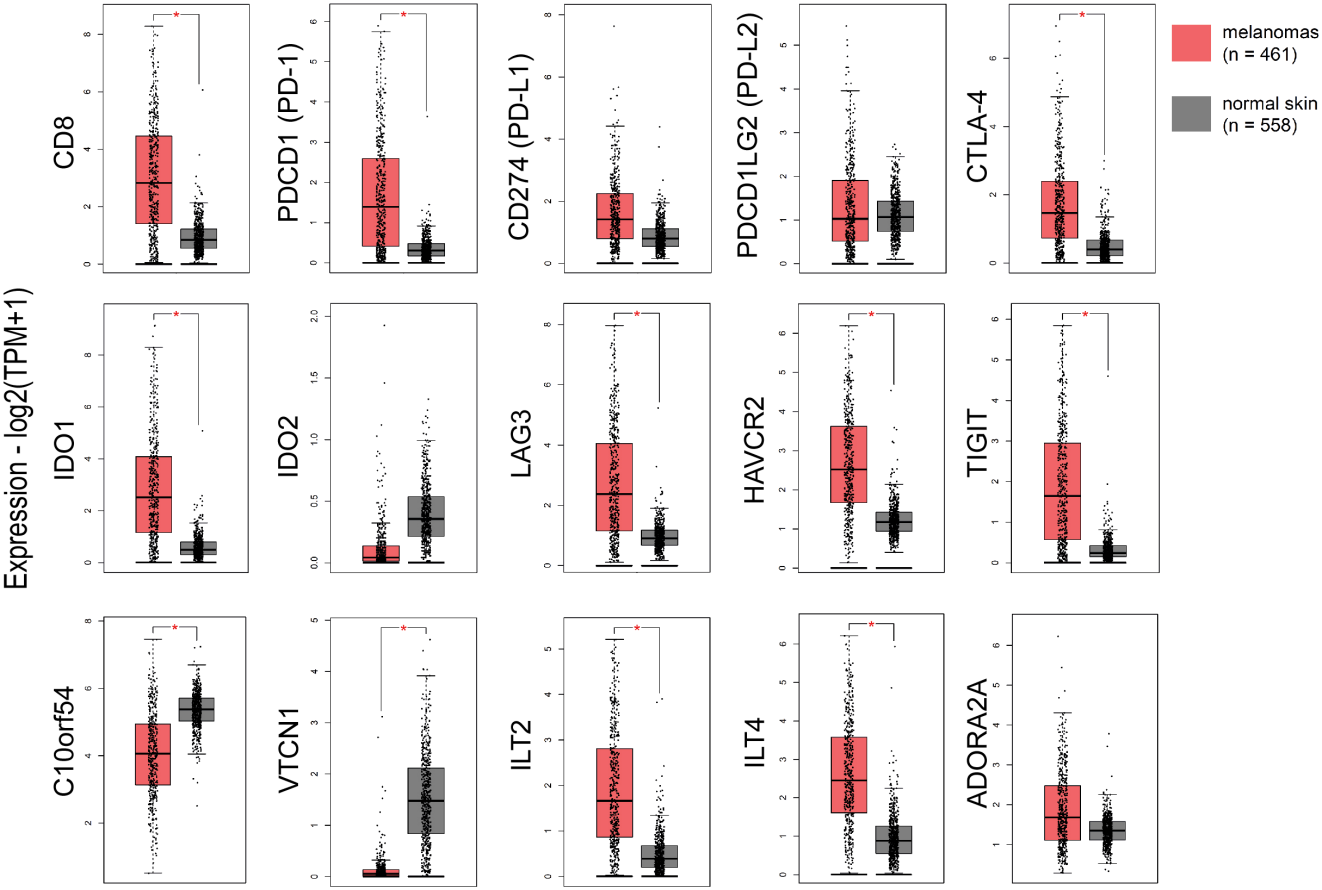
The expression of *CD8*, *PD-1, CTLA-4, IDO1, LAG3, HAVCR2, TIGIT, ILT2* and *ILT4* was significantly higher in skin melanomas; whereas, *C10orf54 (VISTA)* and *VTCN1*, were expressed at markedly lower levels in the tumor samples compared to normal skin samples. Red asterisks (*) denote significant differences (|log_2_FC>1| and p<0.01) between skin melanomas from the TCGA-SKCM dataset and matched normal samples from TCGA and GTEx. One-way ANOVA, using disease state (skin melanoma or normal sample) was used to calculate differential expression. The expression data were first log_2_(TPM+1) transformed for differential analysis and the log_2_FC was defined as median (skin melanoma) - median (normal skin).

**Figure 2:**
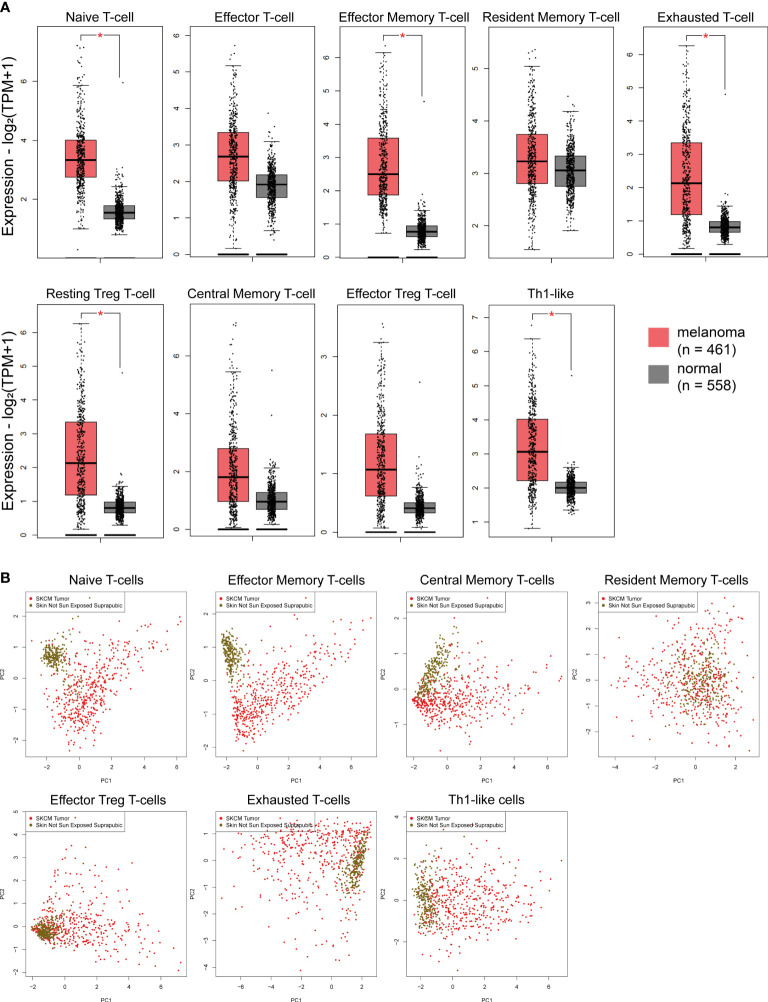
**(A)** Immune-related signatures being upregulated in skin melanomas. The *“naïve T-cell*”, *“effector memory T-cell*”, “*exhausted T-cell*”, “*resting Treg T-cell*” and “*Th1-like*” signatures discriminated best skin melanomas (TCGA-SKCM) from the non-sun-exposed (suprapubic) normal skin (GTEx). Asterisks (*) denote significant differences |Log2FC|>1 and p-value<0.01. **(B)** PCA dimensionality reduction on skin melanoma samples and normal skin tissue not exposed to the sun, based on the expression of each signature.

**Figure 3:**
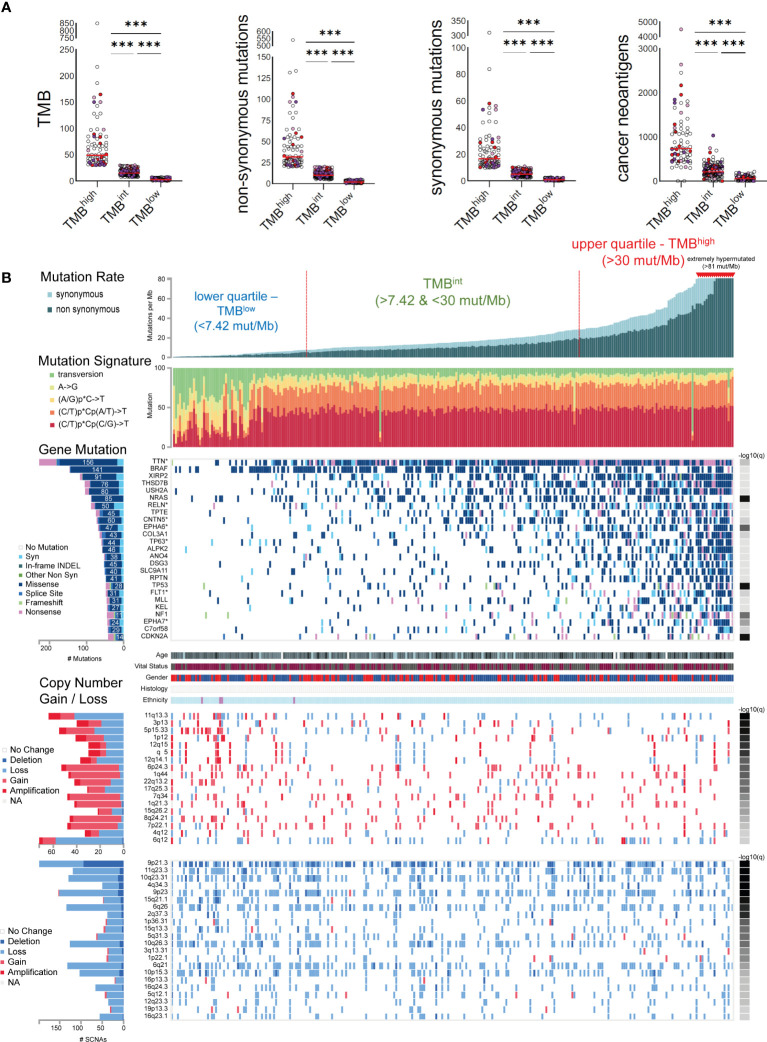
**(A)** Skin melanomas were stratified into upper and lower quartiles. The upper quartile includes TMB^high^ tumors (>30 total mutations/Mb), among which some were extremely hypermutated (>81 total mutations/Mb); whereas the lower quartile contains TMB^low^ tumors (<7.4 total mutations/Mb). Tumors in-between were classified as TMB intermediate (TMB^int^). The scatterplots in the upper part show the total number of mutations (TMB), non-synonymous and synonymous mutations, as well as cancer neoantigens per TMB subgroup. Melanoma samples overexpressing *CD274* (PD-L1) (>2.44 log_2_(TPM+1)) and *CTLA4* (>3.089 log_2_(TPM+1)) are highlighted in red and purple color, respectively. Samples overexpressing both *CD274* and *CTLA4* are colored in light purple. Asterisks (***) denote statistically significant differences in the TMB, non-synonymous mutations, synonymous mutations or cancer neoantigens, between the three subgroups (p<0.0001). **(B)** The mutational signatures differed between TMB^high^ and TMB^low^ tumors, with the first having a preference for (C/T)p*Cp(C/G)>T and (C/T)p*Cp(A/G)>T mutations, whereas the latter, of transversions, A>G and (A/G)p*C>T mutations. The significantly mutated genes include *TTN, BRAF, XIRP2, THSD7B, USH2A, NRAS, RELN, TPTE, CNTN5, EPHA6, COL3A1*, among others. Copy number gains and losses were observed irrespective of the TMB status of the tumors, mainly across 6q12, 11q13.3, 5p15.33, 6p24.3, 9p21.3, 7q34, 11q23.3, 10q23.31, 4q34.3, 9p23 and 6q26.

An independent cohort of 11 skin melanoma samples from the Human Protein Atlas (https://www.proteinatlas.org/) (38) and tissue microarrays (TMAs), containing 40 cases of malignant melanoma, plus 30 adjacent normal skin tissue and 10 skin tissue (ME803b, US Biomax, Inc.) were used to validate protein expression using IHC and evaluate the TIL load with hematoxylin and eosin (H&E) staining. In brief, FFPE sections (4μm) were heated at 50°C overnight. Then, they were deparaffinized in xylene and rehydrated in graded ethanol to distilled water. During hydration, a 5 min blocking for endogenous peroxidase was done in 0.3% H_2_O_2_ in 95% ethanol. Prior to immunostaining, the sections were immersed in 10mM citrate buffer (pH 6.0), rinsed in Tris-buffered saline (TBS) and subjected to heat-induced epitope retrieval (HIER) using a pressure boiler. Sections were then incubated overnight at 4°C with mouse monoclonal antibodies (mAbs) against IDO1 (1:150, Sigma-Aldrich Cat# HPA023149, RRID : AB_1846221), PD-1 (1:250, Sigma-Aldrich Cat# HPA035981, RRID : AB_10669664), PD-L1, a marker specific for T-cells, B-cells and tumor cells (1:50 dilution, clone 22C3, Dako, CA), LAG3 (1:15, Sigma-Aldrich), the cytotoxic T-cell markers CD8A The image used in Figures 1-3 has part labels; however, the description is missing in the caption. Could you clarify this? Provide revised files if necessary.and CD8B (CDA, 1:400 dilution, clone C8/144B, Dako, CA; CD8, 1:100, Sigma-Aldrich Cat# HPA029164), and the Treg-specific marker FOXP3 (1:200 dilution, clone 236A/E7, ThermoFischer Scientific). The UltraVision LP HRP polymer^®^, Ultra V Block and DAB quanto substrate system^®^ (Thermo scientific, CA) were used for detection. Finally, slides were rinsed in tap water, counterstained with hematoxylin, dehydrated in grade ethanol and coverslipped. Slides were then independently assessed by two observers. Sections of hyper-reactive tonsils were used as positive controls for anti-PD-L1 and anti-CD8 staining and preimmune rabbit serum as a negative control for nonspecific staining. Protein staining was scored as 2+ (high (>75% positive cells) or medium (50-75% positive cells) staining), l+ (low staining, 5-25% positive cells) and 0 (staining not detected or <5% positive cells) with strong, medium, weak or negative intensity. The percentage (%) of TILs (200x magnification) in the was also scored. Slide scanning was performed on a VENTANA iScan HT slide scanner v1.1.1 (Roche).

### Somatic mutations in the IFN-γ gene expression signature and immune checkpoint genes

We evaluated gene expression along with the detection of SNVs and Indels across an IFN-γ-related signature, composed of *IDO1, CXCL10, CXCL9, HLA-DRA, STAT1* and *IFNG* (39). We also assessed somatic mutations in the IFN-γ pathway genes *IFNGR1/2, JAK1/2* and *IRF1*, as well as across *BRAF, NRAS, NF1, PTEN* and *B2M* in the TCGA-SKCM dataset. The analysis of somatic mutations was performed using MuTect2 Variant Aggregation and Masking (v.4.1) and gene expression was measured in log_2_(FPKM-UQ+1) values using the UCSC Xena platform (40). MuPIT Interactive (http://mupit.icm.jhu.edu/) was used to map the SNVs on the crystal structure of each protein, in 3D (hg38).

### Detection of immunophenoscores

We calculated IPS scores in TMB^high^ and TMB^low^ tumors (ranging from 0-10) based on the expression of immunomodulators, effector T-cells, effector memory T-cells and immunosuppressors. Their immunophenotypes were visualized using immunophenograms, as previously described (41, 42).

### Patient response to immunotherapy

Tumor Immune Dysfunction and Exclusion (TIDE, http://tide.dfci.harvard.edu/) (43, 44) was used to predict patient response to anti-PD1 or combined anti-PD-1 and anti-CTLA-4 therapy across seven independent skin melanoma datasets (Van Allen et al., 2015 (45), Hugo et al., 2016 (GSE78220) (41), Nathanson et al., 2017 (46), Prat et al., 2017 (GSE93157) (47), Lauss et al., 2017 (GSE100797) (48), Riaz et al., 2017 (GSE91061) (49) and Gide TN, et al., 2019 [PRJEB23709) (50)]. Pre-treatment melanoma tumor expression profiles of patients (log_2_(TPM+1) values) were downloaded and normalized towards the control samples. Each gene was normalized by subtracting the expression value in the reference control samples. Higher TIDE values indicate that the patient has higher potentials of tumor immune evasion and is, therefore, less likely to benefit from the corresponding immune-checkpoint blockade. The IFNG values indicate the IFNγ response biomarkers of *IFNγ*, *ACAT1*, *IDO1*, *CXCL10*, *CXCL9* and *HLA-DRA*. From the analysis we also deduced the expression of CD274 (PD-L1), the average expression from CD8A and CD8B genes, the levels of cytotoxic T-lymphocytes, each patient’s dysfunction of the tumor, exclusion potential of the tumor, as well as the Pearson’s correlation between gene expression and MDSCs, CAFs and M1/M2 TAMs.

### Statistical analysis

Differences in gene expression between high and low activating (or inhibitory) immune receptor-expressing tumors or between TMB^high^ and TMB^low^ tumors, were assessed using the nonparametric Mann-Whitney test. Gene expression (log_2_(TPM+1) values were profiled using violin plots across different pathological stages of the tumors. Multivariate analysis of variance (MANOVA) with the F statistic was used to estimate differences across the different stages. We used Kaplan-Meier curves to plot overall and disease-free survival in patients with high or low expression of immune checkpoints or different multi-gene signatures, using the median expression as cut-off. The log-rank test with HR and 95% CI was used for analysis. Adjusted p-values <0.05 were considered statistically significant. Correlations between each patient’s TIL or TMB load and the expression of immune receptors were assessed using Pearson’s test. All statistical analyses were performed using GraphPad Prism v9.0.0.121. Clusters of similar GO terms were computed using the Leiden algorithm (51) and points were plotted on the first two UMAP dimensions using BokehJS 2.3.2.

## Results

### Deregulated genes and functional analysis in skin melanoma

We initially detected the significantly deregulated genes, having a broad distribution across all chromosomes, in primary and metastatic skin melanoma (**Table S1**), and focused on the top 250 up-/down-regulated genes within each subgroup. The upregulated genes in primary melanomas were enriched in regulation of immune response; cytokine-mediated signaling pathway; antigen receptor-mediated signaling pathway; cellular response to interferon-gamma; regulation of T cell proliferation; regulation of T cell activation; T cell receptor signaling pathway; positive regulation of lymphocyte proliferation; positive regulation of T cell activation; and cellular response to cytokine stimulus (GO-BP), in MHC class II receptor activity; MHC class II protein complex binding; CXCR3 chemokine receptor binding; chemokine activity; and cytokine receptor activity (GO-MF), as well as in MHC class II protein complex; T cell receptor complex; lumenal side of endoplasmic reticulum membrane; and integral component of lumenal side of endoplasmic reticulum membrane, among other GO-CC terms (**Table S2** and **Figure S1**).

On the other hand, the top 250 down-regulated genes were enriched in regulation of extrinsic apoptotic signaling pathway; positive regulation of protein localization to cell periphery; maintenance of protein location in nucleus; response to cytokine; positive regulation of protein localization to plasma membrane; regulation of protein localization to plasma membrane; ribosome biogenesis; and positive regulation of NF-kappaB transcription factor activity, among other GO-BP terms. They were also enriched in cytoskeleton-nuclear membrane anchor activity; chloride channel inhibitor activity; nucleoside-diphosphatase activity; chloride channel regulator activity, among other GO-MF terms, as well as in (cytosolic) large ribosomal subunit; melanosome membrane; and chitosome; pigment granule membrane, among other GO-CC terms (**Table S3** and **Figure S2**).

Among metastatic melanomas, the upregulated genes were enriched in regulation of immune response; cytokine-mediated signaling pathway; antigen receptor-mediated signaling pathway; cellular response to interferon-gamma; regulation of T cell proliferation; regulation of T cell activation; and T cell receptor signaling pathway, among other GO-MF terms. Similar to primary tumors, they were also enriched in MHC class II receptor activity; MHC class II protein complex binding; MHC protein binding; CXCR3 chemokine receptor binding; chemokine activity; and cytokine receptor activity, among other GO-MF terms, as well as in MHC protein complex; MHC class II protein complex; T cell receptor complex; lumenal side of endoplasmic reticulum membrane; and integral component of lumenal side of endoplasmic reticulum membrane, among other GO-CC terms (**Table S4** and **Figure S3**).

Finally, the top 250 down-regulated genes in metastatic melanomas were enriched in the same GO terms as in the primary tumors (**Table S5** and **Figure S4**). Key findings were also validated across four independent GEO datasets (GSE8401, GSE7553, GSE46517 and GSE46517). The top 250 deregulated genes in each dataset were mainly enriched in the GO-BP terms epidermis & skin development, keratinocyte & epidermal cell differentiation, among others.

### High expression of immune-checkpoints associates with the TIL load and can be used as a prognostic marker in melanoma

Focusing on immune checkpoints, we found higher expression for *PD-1, PD-L1, CTLA-4, IDO1, LAG3, HAVCR2, TIGIT* and *ILT4*, as well as for *CD8* in skin melanomas against the normal samples, reflecting the immunosuppressive TME in these tumors. On the other hand, *VISTA* and *VTCN1* were downregulated in skin melanoma, whereas, *IDO2*, *PD-L2* and *ADORA2A* did not differ between melanomas and normal samples (**Figure 1**). Interestingly, the expression of *CD8* and the given immunoreceptors, did not differ stage-wise (**Figure S5**).

In addition, skin melanoma patients expressing highly *CD8, PDCD1, CD274, PDCD1LG2, CTLA-4, C10orf54 (VISTA), LAG3, HAVCR2, TIGIT, ILT2, ILT4, ADORA2A, IDO1* and *IDO2* had better overall survival versus low-expressing patients. What’s more, patients with higher levels of *CD8, VISTA, PD-L2, LAG3, ADORA2A, IDO1, IDO2* and *ILT2* had markedly improved disease-free survival, suggesting that their expression can be used as a prognostic marker, with high levels being favorable in melanoma (**Figure S6**).

The TIL load is a predictive biomarker for patient response to anti-PD1/PD-L1 immunotherapy (52). We hypothesized that TILs associate with the expression of further immune checkpoints in the TME. To verify this assumption, we conducted Pearson’s correlation test with the expression of 11 immune receptors and found that, similar to other cancers (53, 54), the TIL load significantly correlates with *TIGIT* (r=0.503, p=0.05), *IDO1* (r=0.545, p=0.037), *LAG3* (r=0.589, p=0.023) and *ADORA2A* (r=0.589, p=0.037) in skin melanomas, irrespective of their mutation rate.

Furthermore, *CD274 (PD-L1)* expression correlated significantly with the rest immune checkpoints (apart from *VTCN1*) in skin melanoma compared to normal skin (not exposed to the sun), especially with *PD-L2, ILT2, HAVCR2* and *TIGIT* (**Figure S7A**). This finding supports previous evidence that immune response is driven by different immunosuppressive mechanisms within the TME in skin melanoma, which could be tackled using combination immunotherapies, especially in metastatic patients (55, 56). Adding to that, *CD8A* expression correlated significantly with *CD274, PDCD1, PDCD1LG2, IDO1, LAG3, ILT2, HAVCR2, TIGIT, ADORA2A* and *ILT4* expression in the tumor compared to unexposed normal skin, reiterating that CD8 expression is of paramount significance for a successful response to ICI therapies (57, 58) (**Figure S7B**).

### Immune-signatures are activated in skin melanomas irrespective of their molecular subtype

Recent evidence shows that immune signatures are associated with disease prognosis. We thus, investigated 9 immune-related gene signatures in skin melanoma against the normal counterparts, and found a significant enrichment in the “naive T-cell”, “effector memory T-cell”, “exhausted T-cell”, “resting Treg T-cell” and “Th1-like” gene signatures. Naïve T cells are precursors for effector and memory T cell subsets (59). Exhausted T cells are dysfunctional and arise during chronic infection and cancer. Their state is defined by poor effector function, sustained expression of inhibitory receptors and a transcriptional state distinct from that of functional effector or memory T cells (60). Resting Treg cells differentiate as activated Tregs after the antigen exposition (61), whereas, Th1-like cells play a role on inflammatory and autoimmune disorders (62).

On the other hand, the “effector T-cell”, “resident memory T-cell”, “central memory T-cell” and “effector Treg T-cell” signatures did not differ significantly between melanoma and normal samples, despite being higher in the former. Effector T-cells steer the immune responses to execute immune functions. While they were initially found to promote immunity, recent studies unraveled negative regulatory functions of effector T-cells in modulating adaptive, but also innate immunity (63). Resident memory T-cells are critical for maintaining antitumor immunity (63), whereas central memory T-cells mediate a faster, stronger, and more effective response to secondary challenge from a pathogen, compared to naive T-cells. As for Treg cells, they are quite heterogeneous with distinct phenotypical and functional subsets. Naïve-like thymus-derived Tregs, once stimulated, can differentiate into effector Tregs and migrate to peripheral tissues to control immune homeostasis (64). Interestingly, none of the above immune signatures differed between BRAF^mut^, NF1^mut^, RAS^mut^ and Triple^WT^ tumors (**Figure S8**). In addition, PCA analysis for the “effector memory T-cell” and “naïve T-cell” signatures discriminated best skin melanomas from the non-sun-exposed (suprapubic) normal skin (**Figure 2**). Collectively, these findings strongly suggest the activation of several immune-related gene signatures in skin melanoma, irrespective of its molecular subtype, reflecting their link with the disease prognosis.

### Mutational signatures causing high TMB associate with UV light exposure and ageing in melanoma

**Figure 4:**
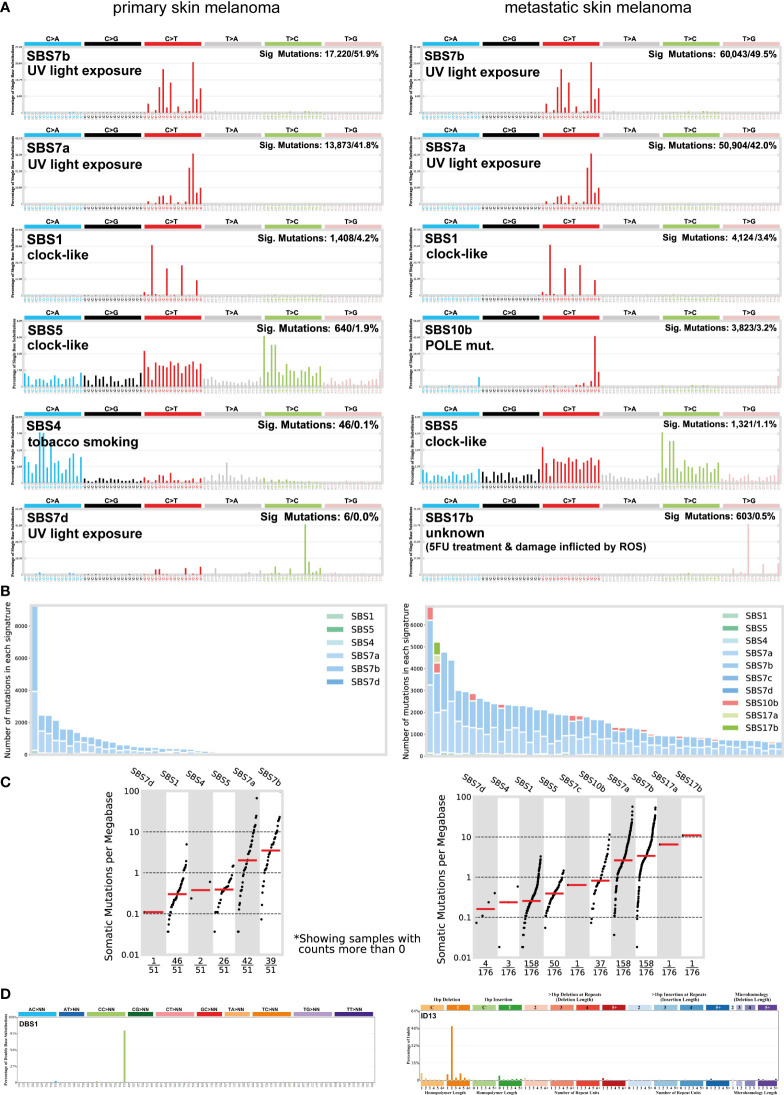
**(A)** The most prevalent single base substitution (SBS) signatures in primary and metastatic skin melanoma. The proposed aetiology of each SBS signature, along with the total number of mutations and corresponding percentage (%) are denoted. SBS signatures were identified using 96 different contexts, considering not only the mutated base, but also the bases immediately 5’ and 3’. **(B)** Activity plots depicting the number of mutations in each signature per skin melanoma patient. **(C)** TMB plots depicting the somatic mutations per Mb. **(D)** The most common doublet base substitutions (DBS) across primary and metastatic skin melanomas, were DBS1 and ID13. DBS signatures were generated after the concurrent modification of two consecutive nucleotide bases.

Skin melanoma patient stratification based on their mutation rate revealed that tumors with >30 mutations/Mb had a different mutational signature profile from those having <7.42 mutations/Mb. The former group was mainly characterized of (C/T)p*Cp(C/G)>T and (C/T)p*Cp(A/G)>T mutations, whereas the latter, of transversions, A>G and (A/G)p*C>T mutations (**Figure 3**). We further analyzed the single-base substitution (SBS) profiles and decomposed each signature to its components and the different percentages of contribution for each of these. As expected, we found that (both primary and metastatic) melanomas were mainly characterized of signatures SBS7a/b (exposure to UV light), SBS1 (spontaneous deamination of 5-methylcytosine; clock-like), SBS5 (clock-like) and SBS10b (*POLE/POLD1* mutations). Interestingly, we found a primary tumor to associate with SBS4 (tobacco smoking) and a metastatic tumor to associate with SBS17b. The latter signature is of unkown aetiology, but previous studies have associated it with 5FU chemotherapy treatment and to damage inflicted by reactive oxygen species (65). As expected, we found ~3.6-times higher number of SBSs among metastatic tumors compared to primary ones (121,175 vs 33,796 SBSs). The mutational signatures exhibiting the highest contribution in primary tumors, were SBS7b (17,220 mutations; 51.9%) SBS7a (13,873 mutations; 41.8%), SBS1 (1,408 mutations; 4.2%) and SBS5 (640 mutations; 1.9%), followed by a small contribution in SBS4 (46 mutations; 0.1%), SBS17b (603 mutations; 0.5%) and SBS7d (6 mutations; 0%). Metastatic tumors on the other hand, were enriched in SBS7b (60,043 mutations; 49.5%) SBS7a (50,904 mutations; 42%), SBS1 (4,124 mutations; 3.4%) and SBS10b (3,823 mutations; 3.2%), followed by a small contribution in SBS5 (1,321 mutations; 1.1%), SBS17b (603 mutations; 0.5%) and SBS17a (357 mutations; 0.3%) (**Figures 4A–C**). Most of these SBSs were previously reported in skin melanoma and their mutational processes are known to cause a high TMB and hypermutation (32, 42, 66–69). As regards *POLE/POLD1* mutated tumors (SBS10b), they have been shown to have a higher number of neoantigens and infiltrating lymphocytes (70).

We also found a substantial variation in the number of doublet base substitutions (DBS) (range, 0-79 DBSs/sample in primary tumors; 0-206 DBSs/sample in metastatic tumors). Among these, we identified a high percentage of DBS1 and ID13, both due to exposure to UV light. DBS1 is mainly composed of CC>TT on the untranscribed strands of genes indicative of damage to cytosine and repair by transcription-coupled nucleotide excision repair (TC-NER), and it associates with SBS7a/SBS7b (71, 72). ID13 is predominantly composed of T deletions at TT dinucleotides, exhibits large numbers of mutations and is also associated with DBS1 (34) (**Figure 4D**). These data reiterate the strong link between UV light exposure with melanoma and ageing.

### Genomic landscape in skin melanomas with diverse TMB

In total, 25 genes were recurrently mutated in skin melanoma, including *TTN* (156 missense out of a total of 228 mutations), *BRAF* (141 missense out of a total of 146 mutations), *XIRP2* (91 missense out of a total of 118 mutations)*, THSD7B* (76 missense out of a total of 105 mutations)*, USH2A* (80 missense out of a total of 104 mutations)*, NRAS* (85 missense out of a total of 88 mutations)*, RLN* (50 missense out of a total of 88 mutations) and *TPTE* (45 missense out of a total of 75 mutations), among others, having a lower mutation frequency (**Figure 3**). As expected, BRAF and NRAS mutations were not common among TMB^high^ patients, as only the BRAF^V600K^ mutation is UV-induced and associates with a higher mutational burden (73). Overall, we identified 40 recurrently mutated cancer drivers, including *BRAF*, *NRAS*, *ARID2* and *TP53*, across 466 tumors within the TCGA-SKCM dataset (413,742 total mutations), among which, *BRAF* dominated (35) (**Table S6** and **Figure S9**). As anticipated, we found differences in the top mutated genes between primary and metastatic skin melanomas, apart from the drivers BRAF, NRAS, TP53 and PTEN, being commonly mutated in the two types (**Figure S9**).

Copy number variations (CNVs) were also observed across all tumor samples, irrespective of their TMB status. In addition, we did not detect any difference in the intra-tumoral genomic heterogeneity between TMB^high^ and TMB^low^ tumors, as reflected by their MATH scores (74). CNVs were mainly located in 6q12 (1.39% deletion, 79% loss, 15.28% gain and 4.17% amplification); 11q13.3 (65.63% loss, 18.75% gain and 15.63% amplification; associated with *WNT11* amplification); 5p15.33 (45.45% loss, 43.64% gain and 10.91% amplification); 6p24.3 (7.55% loss, 84.91% gain and 7.55% amplification); 7q34 (89.58% gain, 4.17% amplification and 6.25% loss; associated with *BRAF* amplification); 8q24.21 (89.13% gain, 6.52% amplification and 4.35% loss; associated with *MYC* amplification); 9p21.3 (47.47% deletion and 52.53% loss); 11q23.3 (7.56% deletion, 92.44% loss); 10q23.31 (10.08% deletion, 89.92% loss; associated with *PTEN* deletion); 4q34.3 (22% deletion, 78% loss); 9p23 (8.50% deletion, 90/20% loss and 1.31% gain), 6q26 (3.73% deletion, 96.27% loss) and 1p22.1 (87.5% loss, 2.5% deletion and 10% gain; associated with *NRAS* reduction) among others (**Figure 3**). These findings are in good agreement with previous reports (75).

### The expression of most immune-receptors is independent of the TMB in skin melanoma

**Figure 5:**
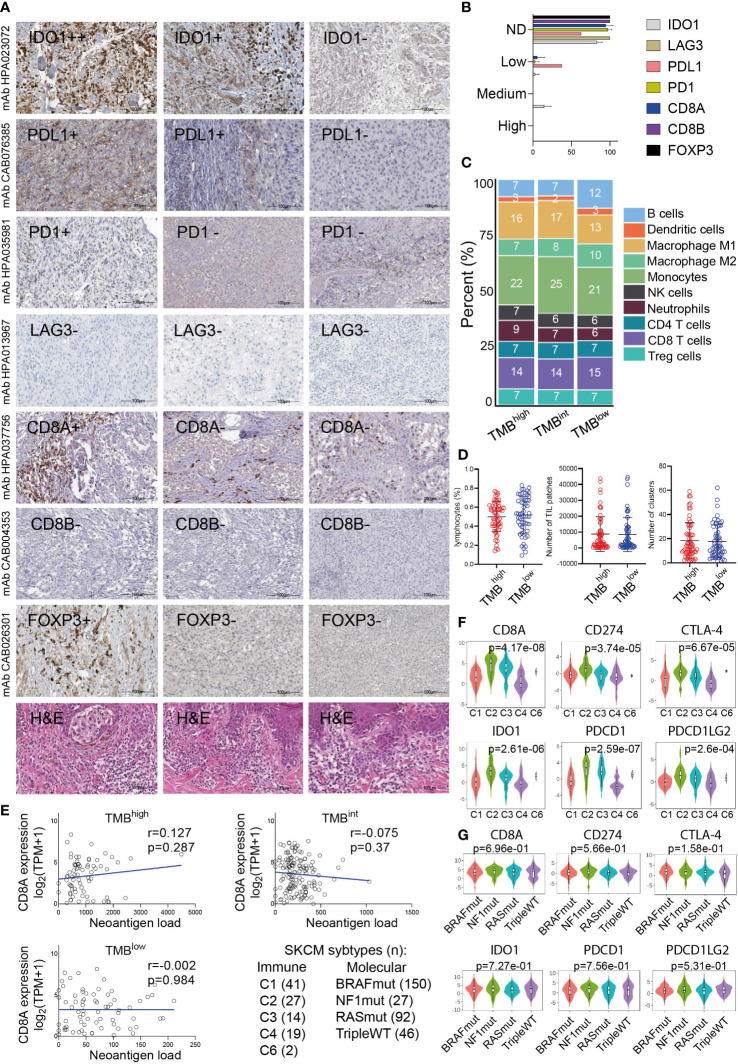
**(A)** Indicative immunohistochemistry (IHC) staining for the inhibitory receptors IDO1, PD-L1, PD-1, LAG3, CD8A/B (marker for cytotoxic T-cells) and FOXP3 (marker for Tregs) in an independent cohort of 11 cutaneous melanomas. H&E, hematoxylin and eosin staining. **(B)** Overall, the protein expression of these markers was either not detected (ND) or low and probably did not differ between TMB^high^ and TMB^low^ tumors. **(C)** Immune-cell fractions across TMB^high^, TMB^int^ and TMB^low^ skin melanomas, using extracted data (quanTIseq) from The Cancer Immunome Database. **(D)** The scatterplots depict the percentage of lymphocytes (%), average number of TIL patches and clusters (with standard deviation, SD) in TMB^high^ (>30 mut/Mb) and TMB^low^ (<7.4 mut/Mb) skin melanomas. Neither of these differed significantly between the two subgroups of tumors. **(E)** The expression of CD8A (log_2_(TPM+1)) did not correlate with the neoantigen load in either TMB subgroup. Expression of CD8A, PDCD1 (PD-1), CD274 (PD-L1), PDCD1LG2 (PD-L2), IDO1 and CTLA-4 across different immune **(F)** and molecular **(G)** subtypes in skin melanoma. Immune subtypes: C1, wound healing (n=41); C2, IFN-gamma dominant (n=27); C3, inflammatory (n=14); C4, lymphocyte depleted (n=19); C5, immunologically quiet (n=0); C6, TGF-b dominant (n=2). Molecular subtypes: BRAF^mut^ (n=150), NF1^mut^ (n=27), RAS^mut^ (n=92), triple^WT^ (n=46).

PD-L1 expression and TMB were recently shown to be independent biomarkers in most cancers (76). Here, we evaluated the expression of *CD274* (*PD-L1*) along with other immunoinhibitors and immunostimulators, across TMB^high^, TMB^int^ and TMB^low^ skin melanomas. Globally, we found that the expression of most immunoreceptors does not differ across the three TMB subgroups (p>0.05) (**Figures S10**–**S13**). *CD274* expressed higher in TMB^high^ tumors (p<0.05), but still without any significant correlation with the TMB (Pearson’s rho (r)=0.052, p=0.372). We also noted differences in the expression of *TNFSF18*, *KDR* and *ENTPD1*, which were lower in TMB^high^ tumors (p<0.05) but also did not correlate significantly with the TMB (*TNFSF18*, r=-0.043, p= 0.459; *KDR*, r=-0.073, p=0.214; *ENTPD1*, r=0.0002, p=0.997). In contrast, the expression of *TNFSF9* was marginally higher in TMB^low^ melanomas (p=0.06) and correlated negatively with the TMB (r=-0.146, p=0.013). A few other correlations we could note, were between *TNFSF9* and TMB (r=-0.146, p=0.013), *NT5E* and TMB (r=0.134, p=0.023), as well as between *MICA* and TMB (Pearson’s r=0.167, p=0.004). Paradoxially, however, the expression of several well-known inhibitory receptors, including *CTLA-4, PDCD1 (PD-1), TIGIT, IDO1, LAG3, ADORA2A* and *VTCN1*, was similar between TMB^high^ and TMB^low^ tumors, corroborating that in general, the expression of immune checkpoints and TMB are independent biomarkers in skin melanoma. This finding was further supported by our IHC data, showing that PD-1, PD-L1, IDO1 and LAG3 protein levels are also similar across melanomas of differential TMB status (**Figures 5A, B**). In addition, PD-L1+ cells (when expressed) were topologically associated with CD8+ T-cells. The TIL percentage (%) also, did not differ significantly across the three TMB subgroups of tumors (TMB^high^, 1.77 ± 2.63; TMB^int^, 2.74 ± 5.46; TMB^low^, 1.72 ± 3.03); it was higher in the stroma than in the parenchyma of primary tumors, but this percentage decreased in the metastatic cases. Taken together, these data suggest that TMB is not the only factor that affects immunogenicity. In fact, other factors apart from high PD-L1 expression, seem to also affect immunogenicity in skin melanoma and therefore, prevent TMB^low^ patients to benefit from ICI therapies. These include high levels of IFNγ, CD8 and GZMA/PRF1 [intra-tumoral immune cytolytic activity (23, 42, 77)], as well as low levels of MDSCs, CAFs or M2 macrophages in the TME.

To further investigate these factors, we examined the fractions of 10 immune cell types, including B-cells, DCs, M1/M2 macrophages, NK cells, neutrophils, CD4+, CD8+T-cells and Tregs, among the three TMB subgroups of melanomas. Interestingly, we observed a similar immune-cell fraction between TMB^high^ and TMB^int^ tumors, both having a higher ratio of M1/M2 macrophages compared to TMB^low^ tumors. In addition, the CD8+ T-cells/Tregs ratio was similar between the three TMB subgroups (**Figure 5C**). Other than this, the total number of lymphocytes and the rest immune cells did not differ between TMB^high^ and TMB^low^ melanomas, neither did the number of TIL patches or clusters that they formed (**Figure 5D**), suggesting the existence of other mechanisms allowing or inhibiting response to ICI therapies. These findings also agree with the notion that the content of CD8+ cytotoxic T cells within the TME, along with the TMB, are both crucial factors in determining patient resistance to ICI therapies. In line with this, McGrail et al. showed that CD8+ T-cell levels positively correlate with the neoantigen load in melanoma and that TMB^high^ tumors have a better response to ICI compared to TMB^low^ ones (78). Nevertheless, in terms of CD8A gene expression, our data show that this does not correlate with the neoantigen load in either TMB subgroup (**Figure 5E**). As regards the number of TIL clusters in different molecular subtypes of skin melanoma, this was recently evaluated in the same cohort and it was found to associate with better survival in BRAF^V600E/K^ patients, but neither in NRAS^mut^ nor BRAF^wt^/NRAS^wt^ patients (79). We also found that CD8A, PDCD1 (PD-1), CD274 (PD-L1), PDCD1LG2 (PD-L2), IDO1 and CTLA-4 are highly expressed in the ‘IFN-gamma dominant’ (C2) and ‘inflammatory’ (C3) immune subtypes, but not across the different molecular subtypes (BRAF^mut^, NF1^mut^, RAS^mut^ or triple^WT^) (**Figures 5F, G**).

### Mutations in the IFNγ pathway could affect immunogenicity in melanoma patients

**Figure 6:**
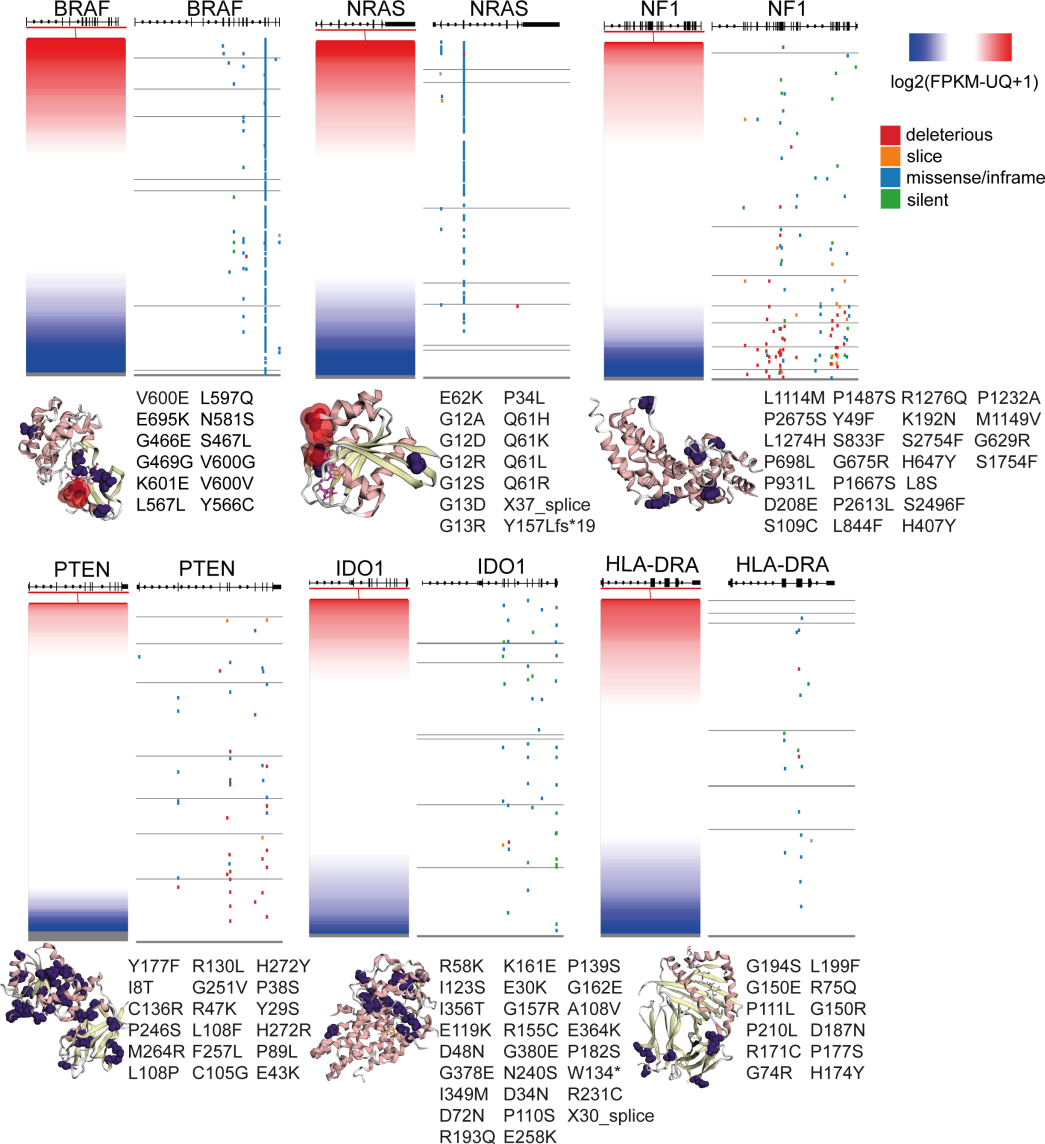
Gene expression and somatic mutations (deleterious, slice, missense/inframe or silent) in the genes BRAF, NRAS, NF1, PTEN, IDO1 and HLA-DRA in skin melanoma. The crystal structures (3D) of the genes’ encoded proteins along with their somatic mutations detected in the TCGA-SKCM dataset (purple color), were calculated using MuPIT (hg38 coding) and are depicted on the right of each plot. Hotspot BRAF and NRAS mutations are highlighted in red color in the corresponding crystal structures. Apart from BRAF, NRAS, NF1 and PTEN, all of which are well-known to be recurrently mutated in skin melanoma, IDO1 and HLA-DRA were also significantly mutated, but the somatic mutations did not seem to affect their protein expression.

IFNγ-related gene expression signatures have been shown to predict patient response to PD-1 checkpoint blockade in melanoma (39). Motivated by these observations, we hypothesized that mutations in the IFNγ pathway could also affect immunogenicity in melanoma patients, apart from the high IFNγ levels. Therefore, we explored the mutational pattern of genes in the IFNγ pathway signaling, to find whether they associate with T-cell insensitivity, and therefore, resistance to immunotherapy. Notably, we found an increased number of SNVs in *IDO1* and *HLA-DRA* (MHC-II protein). In specific, these contained 28 missense mutations, 1 stop gained and 1 splice acceptor variant in *IDO1* (**Figure 6** and **Table S7**), which however did not seem to disturb the gene’s expression, as they did not affect the protein’s, heme-ring. Therefore, the ability of IDO1 to catalyze the deoxygenation of tryptophan does not seem to be affected. Kynurenine is the metabolic product of tryptophan, which suppresses T-cell proliferation and promotes the development of Treg cells. Its inhibition could be exploited therapeutically in cancer immunotherapy beyond ICI or adoptive transfer of chimeric antigen receptor (CAR) T-cells, since it may restore T-cell function and reduce the accumulation of Tregs (80, 81).

In *HLA-DRA*, we detected 14 missense variants, corroborating the dynamic role of the function of MHC in the progression of the disease (82). HLA-II expression has also been shown to predict patient response to anti-PD1, but not to anti-CTLA-4 immunotherapy (82). HLA-DRa also exhibited heterogenous expression in melanoma lesions and cell lines, with IFNγ being a strong inducer of HLA class II expression (83).

In addition, we noted 3 missense mutations in *CXCL10* and 5 missense mutations, one 5’ UTR and one stop gained variant in *CXCL9*, 6 missense mutations in *STAT1*, as well as 5 missense mutations in *IFNG*, including 1 splice donor, 1 stop-gained, three 5’ UTR variants and one 3’ UTR variant (**Figure 6** and **Table S7**).

As expected, *BRAF* and *NRAS* were the most frequently mutated genes among all patient samples, hosting hotspot mutations (274 missense mutations, 2 in frame deletions and one 3’ UTR variants in *BRAF*; and 121 missense mutations, one frameshift and one splice donor variant in *NRAS*), followed by *NF1* (34 missense, 30 stop gained, 5 frameshift, one 3’ UTR, 3 splice acceptor and 1 splice donor variants and 2 splice region;synonymous variants) and *PTEN* (23 missense, 14 frameshifts, 1 in frame insertion, 4 splice donor/acceptor variants and 6 stop gained mutations). Finally, we detected a smaller number of somatic mutations in *B2M* (1 in frame deletion, 2 splice donor and 1 coding sequence variants), *IFNGR1* (4 missense and two 3’ UTR variants), *IFNGR2* (1 missense, one 3’ UTR and one splice region variant in chr21), *JAK1* (10 missense mutations, one 5’ UTR and 1 stop gained variant), *JAK2* (2 frameshift and 6 missense mutations) and *IRF1* (1 missense and one 3’UTR variant) (**Table S7**). Apart from the activating *NRAS* mutations (linked with high NRAS expression) and the inactivating *NF1* mutations (linked with decreased NF1 expression), all the other mutations were randomly distributed across all melanoma tumors, irrespective of their gene expression (**Figure 6**). Collectively, these data show that mutations in the IFNγ pathway could affect immunogenicity in melanoma patients.

### Patient response to ICI therapies is independent of their TMB status

**Figure 7:**
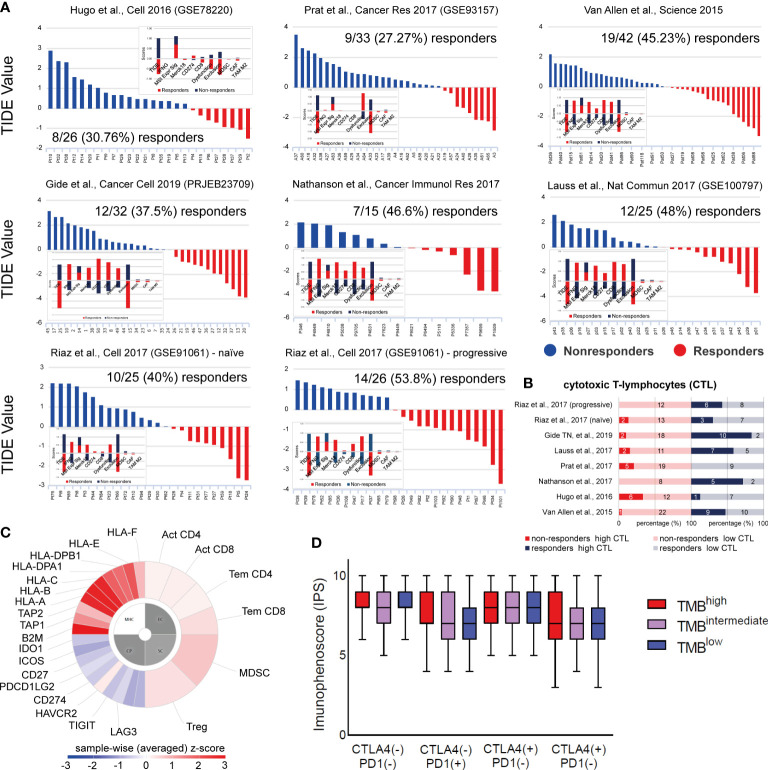
**(A)** TIDE analysis was used to predict patient response to ICI in 7 independent datasets of skin melanoma patients. Higher TIDE score (blue) denotes non-responders to immune-checkpoint blockade, whereas lower TIDE score (red) denotes responders. **(B)** Percentage (%) of high or low cytotoxic T-cell lymphocytes (CTLs) among responders or non-responders to ICI therapies, across all 7 melanoma datasets. Clearly, higher CTL levels were found among ICI-responders. The numbers of ICI-responders or non-responders with high or low CTL levels, are indicated within each bar. **(C)** Indicative immunophenogram depicting the four categories of markers, the expression of which, was used to calculate the immunophenoscores (IPS) for each TMB subgroup of patients. These include: MHC molecules (MHC), immunomodulators (CP), effector cells (EC) and suppressor cells (SC). The outer part of the circle includes individual factors; whereas, the inner part illustrates the weighted average z-scores of the factors included in each category. Sample wise z-scores were positively weighted according to stimulatory cells and negatively weighted according to inhibitory cells and averaged. **(D)** The boxplots indicate the average IPS across the three TMB subgroups, treated with combination ICI therapy [CTLA-4 (+)/PD1 (+)] or with each ICI therapy, alone [CTLA-4 (+)/PD1 (–) or CTLA-4 (–)/PD1 (+)] or none [CTLA-4 (-)/PD1 (-)]. Similar IPS scores were found across all tumors, suggesting that ICI therapy can be applied independently of the patient’s TMB status.

Tumor immune evasion is based on the infiltration of dysfunctional T-cells in the tumor, but also the prevention of T-cell infiltration into the TME. TIDE scores predict better patient response to anti-PD-1 and anti-CTLA-4 therapies, compared to TMB and PD-L1, and can be used to predict longer patient overall survival (84). Using 7 publicly available transcriptome profiles of ICI-treated melanoma patients, we predicted their response based on their TIDE scores. Overall, patient response rate to ICI ranged between 27-53%, depending on their number in each dataset and the ICI therapy given. Broadly, non-responders (high TIDE score) had significantly lower IFNG, Merck18, CD274 (PD-L1), CD8 and ‘dysfunction of the tumor’ scores. In contrast, they had higher ‘exclusion potential of the tumor’ scores, as a result of the higher levels in MDSCs, CAFs and M2 macrophages. As expected, microsatellite instability (MSI) did not associate with melanoma patient response to ICI therapies, obviously due to its low prevalence in this tumor type. Importantly, we found higher CTL levels among ICI-responders compared to non-responders (**Figures 7A, B**), recapitulating previous findings (85).

Next, we questioned whether the TMB status associates with the outcome of each ICI therapy. Therefore, we calculated the immunophenoscores between TMB^high^, TMB^int^ and TMB^low^ patients treated with anti-PD1 or anti-CTLA-4 alone, a combination of both immune checkpoint inhibitors, or any of them. Interestingly, our analysis revealed similar IPS scores across all TMB subtypes (**Figures 7C, D**), suggesting that ICI therapy is independent of the patient’s TMB status alone, and it could thus, also work effectively to treat TMB^low^ patients. Our data also clearly point that the quality of mutations is a more important factor than their quantity, in terms of their immunologic impact on patient response to ICI therapy.

## Discussion

In the present study, we explored the expression of various activating or inhibitory immunoreceptors in skin melanomas with diverse TMB, and evaluated their association with patient survival and the TIL load. Overall, our findings show that high expression of most immunoreceptors, apart from PD-1, PD-L1/L2 and CTLA-4 that have been already tested in the clinical setting, associates with the TIL load and patient survival, but not with the TMB, in contrast to other, less hypermutated and/or non-inflamed tumor types (24, 53).

High TMB was initially noted to correlate with response to anti-CTLA-4 immunotherapy in melanoma (45, 86). During the next years, TMB was employed in many clinical trials of anti-PD-1/PD-L1 agents for treating various cancer types. Patients with higher TMB tended to exhibit better treatment response, but the testing methods and cutoffs of TMB varied across trials (45, 87, 88). In contrast to the widely accepted threshold of ≥10 mut/Mb to define TMB^high^ tumors, in our study we used a more stringent criterion, setting this threshold in the upper 25^th^ quartile (≥30 mut/Mb, TMB^high^), but we also defined as TMB^int^ those tumors with a mutational burden between 7.42 and 30 mut/Mb.

Overall, our findings suggest that TMB^high^ skin melanomas correlate with high levels of IFNγ, CD8+ T-cells in the TME, cancer neoepitopes, as well as high expression of PD-L1 and further immune receptors. In addition, TMB^high^ patients experience longer survival and greater response rates after ICI therapy, compared to TMB^low^ ones (89). The number of cytotoxic CD8+ T-cells modulates immunogenicity in the TME. CD8+ T-cells are the most powerful effectors during an anticancer immune response and constitute the backbone of cancer immunotherapy (90, 91). TMB^high^ skin melanomas also correlate with intratumoral immune cytolytic activity (CYT), defined by the expression of granzyme A and perforin 1, both secreted by effector cytotoxic CD8+ T-cells and NK cells against their target cells (72, 87). CYT is significantly elevated upon CD8+ T-cell activation, as well as during a productive clinical response against immune-checkpoint blockade therapies in melanoma patients (23). The presence of several immune-exclusive cells in the TME, such as MDSCs, CAFs and M2 macrophages also affects response to ICI therapies (57).

By stratifying patients based on their TMB, we found that those having a higher mutation rate (>30 mut/Mb) did not express higher *CTLA-4, PD-1, IDO1* or other immunoreceptors, apart from just a few cases (including *CD274* which was upregulated in TMB^high^ tumors). In contrast, *TNFSF18*, *KDR* and *ENTPD1* showed lower expression levels among TMB^high^ tumors, and also did not correlate with the TMB. Collectively, these observations strongly indicate that immunogenicity in these tumors is affected by other factors as well, other than the TMB, and therefore, TMB^low^ patients could also benefit from ICI therapies.

In the KEYNOTE-002 study, pembrolizumab (anti-PD-1) was established as a new standard treatment after progression on ipilimumab (anti-CTLA-4) and other therapies (92). A year later, in the KEYNOTE-006 study, pembrolizumab was shown to prolong progression-free survival and overall survival and had less high-grade toxicity compared to ipilimumab in patients with advanced melanoma (93). In the CheckMate-066 study, nivolumab was also shown to associate with significant improvements in overall survival and progression-free survival, as compared with dacarbazine, among previously untreated metastatic melanoma patients, without a BRAF mutation (94). Similar improvements associated with ICI therapies were reported elsewhere (13, 95).

Our findings also corroborate that the expression of immune checkpoints and the quantification of the mutational burden seem to be independent predictive biomarkers of ICI therapy in melanoma patients. These results are in line with recent reports mentioning that PD-L1 expression and TMB have non-overlapping effects on the response rate to PD-1/PD-L1 inhibitors and can thus, be used to categorize the immunologic subtypes of different tumor types (76, 96). In addition, despite that TMB associates with improved treatment response, the mutation frequency in expressed genes was found to be superior in predicting the outcome. Additionally, the pre-existing T- and B-cell immunity was shown to play a key role in therapeutic outcomes (97).

We also show that, apart from CTLA-4 and PD-1, there are many other immune receptors expressed by T-cells, which influence the TME and act as checkpoints, negatively regulating immune responses in skin melanoma (24, 98). As combination ICI therapy has been proven to provide clinical benefits for patients with advanced metastatic melanoma, as in other cancer types (99, 100), our data further open up new perspectives for combining the currently administered immune checkpoint blockers, ipilimumab, nivolumab and pembrolizumab with mAbs towards additional inhibitory molecules. These include IDO1, IL2RA, TIGIT, LTA, VTCN1, TIM3, KDR, ENTPD1 and LAG3, as well as agonistic mAbs targeting activating immune receptors, such as TNFSF18, CD70, ICOS and KLRK1. In this line, FDA recently approved the combination therapy of nivolumab (anti-PD-1) and relatlimab (anti-LAG-3 mAb, Opdualag), which was shown to provide a greater benefit with regard to progression-free survival than inhibition of PD-1 alone, in patients with previously untreated metastatic or unresectable melanoma (REALITIVY-047, ClinicalTrials.gov number, NCT03470922) (101).

Importantly, we show that the TIL load is significantly higher among TMB^high^ skin melanomas, providing evidence that patients with a high number of immunogenic mutations have an increased survival. Indeed, the lymphocytic score associated with better survival in these patients, in agreement with previous reports (102, 103). The tumors also had higher CTL numbers, as deduced from their *CD8A* expression. To examine further the factors that could contribute to treatment response or resistance among melanoma patients receiving anti-PD-1 and/or anti-CTLA-4 immunotherapy, we evaluated transcriptomic data from 7 independent datasets and found that indeed, the number of CTLs in the TME associates with patient response to ICI therapy, irrespective of the patient TMB status.

In addition, we investigated different immune-related gene signatures. We found several T-cell-related signatures, including those of naive T-cells, effector memory T-cells and exhausted T-cells, all being upregulated in skin melanoma compared to the normal skin (or matched blood). Other signatures involving inhibitory cells (effector Treg T-cell and resting Treg T-cell signatures), or helper T-cells (Th1-like cell signature), were also higher in skin melanoma, underlying the intricate immunological reactions taking place within the tumor’s microenvironment. Looking deeper into the fractions of immune-cells within the TME however, we did not observe significant differences between TMB^high^ and TMB^low^ tumors, apart from the ratio of M1/M2 macrophages, which was higher in the TMB^high^ subgroup.

Notably, different genomic events and the immune microenvironment in skin melanoma seem to orchestrate the patients’ resistance to ICI therapies or their relapse (42). Frameshift mutations, indels and splice-site mutations are also believed to generate more immunogenic neoantigens compared with the nonsynonymous SNVs that are more frequently detected upon TMB assessment (104). In addition, cancer neoantigens that are similar to pathogen-derived antigens can affect tumor immunogenicity and thus, patient response to ICI therapy (86). We explored the SNVs and CNVs across the different TMB subgroups of tumors, and also highlighted the mutational signatures contributing more to this mutational burden. Chronic sun exposure over years permits the accumulation of sun damage, and it correlates with the age of melanoma diagnosis. Therefore, is was expected to observe mainly UV-light (SBS7a/b/d) and clock-like signatures (SBS1 and SBS5) across all melanomas. In addition, we found that a small percentage of these tumors also associated with *POLE/POLD1* mutations (SBS10b) and tobacco smoking (SBS4).

Together with granzyme B and perforin, IFN-γ acts as a cytotoxic cytokine that initiates apoptosis in tumor cells (105, 106). IFN-γ also enables the synthesis of immune checkpoint inhibitory molecules and indoleamine-2,3-dioxygenase (IDO), thus stimulating other immune-suppressive mechanisms (107–109). The IFN-γ signaling pathway enhances MHC expression and subsequent tumor antigen presentation. It also induces the recruitment of further immune cells, and inhibits tumor cell proliferation (110). IDO1 associates with adverse clinical outcome in melanoma patients, and its activity promotes an immunosuppressive TME by upregulating trafficking of MDSCs and Tregs (111). Here, we evaluated somatic mutations in *IFNG* and other IFN-γ-related genes in skin melanoma, and questioned whether their presence associates with gene expression. Our data reveal that *IDO1* and *HLA-DRA* are frequently mutated in skin melanoma, but these mutations do not seem to associate with their gene expression. Nevertheless, the frequency of the somatic mutations that we detected both in *IDO1* and *HLA-DRA*, suggests that these are common events taking place in skin melanoma and could be involved in hindering patient response to ICI therapies. Their contribution to immune evasion and resistance to ICI therapies, could take place in parallel with other well-known mutations in *BRAF*, *NRAS*, *NF1*, *PTEN* and *B2M*, as well as in other genes involved in the IFN-γ signaling pathway, being critical in mediating antitumor immunity (112).

We finally showed that non-responders to anti-PD-1 and/or anti-CTLA-4 ICI therapies have lower IFNG, Merck18, CD274 and CD8 scores, and lower dysfunction of the tumor. In addition, they have higher exclusion potential of the tumor and higher levels in the immune suppressive MDSCs, CAFs and M2 macrophages, compared to ICI-responders. The latter cell types, on their own and cooperatively, induce an immune-suppressive TME that prevents anti-tumor cytotoxic and Th1-directed T-cell activities, mainly through the release of cytokines, chemokines, and other soluble mediators (113). In addition, their depletion increases anti-tumor immune responses overcoming innate resistance (114). Non-responders to monotherapy often express alternate immune-checkpoints, such as IDO1, ICOS, and TIGIT, in contrast to combination therapy on non-responders, who rarely express these alternate drug targets (50). Moreover, ICI responders had significantly higher CTL numbers compared to non-responders. Therefore, it seems that IFNɣ-associated genes and CTLs in the TME, along with a high TMB (and consequently neoantigen) load, but no specific gene mutation, associate with ICI therapy response. These data provide important insights to facilitate the development of precision immuno-oncology for skin melanoma patients.

Overall, we highlight the associations between various immune receptors, TMB, TILs, patient survival and their response to ICI therapies. Taken together, our data highlight the importance of pre-existing T-cell immunity in the therapeutic outcome. They also corroborate that the expression of most immunoreceptors and TMB are independent biomarkers in predicting treatment response in skin melanoma and that ICI therapies could also be applied to TMB^low^ patients.

## Data availability statement

The original contributions presented in the study are included in the article/**Supplementary Material.** Further inquiries can be directed to the corresponding author.

## Author contributions

GG and AZ acquired data and analyzed them. AZ developed the methodology, analyzed data and interpreted them. AZ wrote the manuscript and supervised the study. GG and AZ critically reviewed the manuscript. All authors contributed to the article and approved the submitted version.

## Acknowledgments

We would like to acknowledge TCGA, ICGC, HPA, GDAC and TCIA for providing the genetic and clinical data of the skin melanoma patients that were analyzed in this study. We would also like to acknowledge assistance with extracting mutational signatures from Dr. Ilias Georgakopoulos-Soares.

## Conflict of interest

The authors declare that the research was conducted in the absence of any commercial or financial relationships that could be construed as a potential conflict of interest.

## Publisher’s note

All claims expressed in this article are solely those of the authors and do not necessarily represent those of their affiliated organizations, or those of the publisher, the editors and the reviewers. Any product that may be evaluated in this article, or claim that may be made by its manufacturer, is not guaranteed or endorsed by the publisher.

## Supplementary material

Supplementary Figure 1 The bar charts (left) depict the top 10 enriched Gene Ontology (GO) terms in the top 250 upregulated genes in primary skin melanoma, along with their corresponding p-values. Asterisks (*) indicate the terms with significant adjusted p-values (<0.05). The scatterplots (right) were created using UMAP and are organized so that similar gene sets are clustered together. Larger, black-outlined points represent significantly enriched terms, the associated gene set names and p-values of which, are denoted.

Supplementary Figure 2 The bar charts (left) depict the top 10 enriched Gene Ontology (GO) terms in the top 250 downregulated genes in primary skin melanoma, along with their corresponding p-values. Colored bars correspond to terms with significant p-values (<0.05). Asterisks (*) indicate the terms with significant adjusted p-values (<0.05). The scatterplots (right) were created using UMAP and are organized so that similar gene sets are clustered together. Larger, black-outlined points represent significantly enriched terms, the associated gene set names and p-values of which, are denoted.

Supplementary Figure 3 The bar charts (left) depict the top 10 enriched Gene Ontology (GO) terms in the top 250 upregulated genes in metastatic skin melanoma, along with their corresponding p-values. Colored bars correspond to terms with significant p-values (<0.05). Asterisks (*) indicate the terms with significant adjusted p-values (<0.05). The scatterplots (right) were created using UMAP and are organized so that similar gene sets are clustered together. Larger, black-outlined points represent significantly enriched terms, the associated gene set names and p-values of which, are denoted.

Supplementary Figure 4 The bar charts (left) depict the top 10 enriched Gene Ontology (GO) terms in the top 250 downregulated genes in metastatic skin melanoma, along with their corresponding p-values. Colored bars correspond to terms with significant p-values (<0.05). Asterisks (*) indicate the terms with significant adjusted p-values (<0.05). The scatterplots (right) were created using UMAP and are organized so that similar gene sets are clustered together. Larger, black-outlined points represent significantly enriched terms, the associated gene set names and p-values of which, are denoted.

Supplementary Figure 5 Stage-plot analysis of the expression of *CD8* and several immune receptors in skin melanoma, showing no significant differences according to the tumor’s stage.

Supplementary Figure 6 The Kaplan-Meier curves show the overall and disease-free (DF) survival of melanoma patients, expressing high or low expression levels of *PD-1, PD-L1/L2, CTLA-4, LAG3, IDO1/2, TIGIT, HAVCR2, VISTA, VTCN1, ILT2/4, ADORA2A* and *CD8* (marker for CD8+ T cells). The log-rank test was used to assess statistical differences between the two subgroups of patients. The patients were separated into high expression group (upper 50 percentile, red curve) and low expression group (lower 50 percentile, blue curve) by gene expression levels. The numbers of the patients in each group are provided as “n(high)” and “n(low)”, respectively. The log-rank p-value, along with the HR(high) and p(HR) values are also provided in each Kaplan-Meier survival plot. A Bonferroni-corrected cut-off log-rank p-value of <0.05 indicates statistical significance.

Supplementary Figure 7 The scatterplots depict the Pearson’s correlation coefficient (R and p-values) between the expression of *CD274 (PD-L1)* **(A)** or *CD8A* **(B)** and various immune receptors in skin melanomas (TCGA-SKCM) and normal suprapubic skin sample, not exposed to the sun (GTEx).

Supplementary Figure 8 The boxplots depict nine immune-signatures which did not differ across BRAF^mut^, NF1^mut^, RAS^mut^ and Triple^WT^ skin melanoma tumors. Signatures were calculated in log_2_(TPM+1) using the |log_2_FC>1| and p<0.01 (ANOVA) as thresholds for statistical significance across the different skin melanoma subtypes.

Supplementary Figure 9 The comutation plots depict the top 20 significantly mutated genes (SMGs, FDR<0.1) in primary and metastatic **(A)** or TMB^high^ and TB^low^ skin melanomas **(B)**. Green, red, pink, black and orange boxes indicate missense, nonsense, translation start site, multi-hit and splice-site mutations, respectively. The SMGs that correlate with primary or metastatic tumors (p<0.05) are highlighted by red or blue circles, respectively, next to the gene names. Each SMG’s q-values (−log10(FDR)) are plotted as a right-side bar plot in blue color. **(C)** The bar chart depicts the top 30 cancer drivers in skin melanoma. **(D)** The lollipop plots (below) report all the variants affecting the coding region of three drivers in skin melanoma (BRAF, NRAS and ARID2). Diagram circles are colored with respect to the corresponding mutations. Passenger mutations are highlighted in light blue and ad driver mutations in red.

Supplementary Figure 10 The scatterplots show mean values in log_2_(TPM+1) with standard deviation (SD) of gene expression across various immunostimulators. Gene expression did not change across TMB^high^, TMB^int^ and TMB^low^ skin melanomas, apart from *CD274* and *TNFSF18* (*, p<0.05. **, p<0.01).

Supplementary Figure 11 The scatterplots show mean values in log_2_(TPM+1) with standard deviation (SD) of gene expression across various immunoinhibitors. Gene expression did not change across TMB^high^, TMB^int^ and TMB^low^ skin melanomas, apart from *KDR*, which was lower in TMB^high^ tumors (**, p<0.01).

Supplementary Figure 12 Pearson’s correlation between TMB and the expression of activating immune receptors (immunostimulators), shows that there was no relationship between their gene expression and the TMB in skin melanoma.

Supplementary Figure 13 Pearson’s correlation between TMB and the expression of inhibitory immune receptors (immunoinhibitors), shows that there was no relationship between the gene expression and the TMB in skin melanoma.

Supplementary Table 1 Significantly up- and down-regulated genes in skin melanoma (TCGA-SKCM).

Supplementary Table 2 Gene Ontology enrichment for the top 250 up-regulated genes in primary skin melanoma.

Supplementary Table 3 Gene Ontology enrichment for the top 250 down-regulated genes in primary skin melanoma.

Supplementary Table 4 Gene Ontology enrichment for the top 250 up-regulated genes in metastatic skin melanoma.

Supplementary Table 5 Gene Ontology enrichment for the top 250 down-regulated genes in metastatic skin melanoma.

Supplementary Table 6 Recurrently mutated cancer drivers in skin melanoma, identified using IntOGen analysis.

Supplementary Table 7 Somatic mutation analysis for IDO1, HLA-DRA, CXCL10, CXCL9. STAT1, IFNG, B2M, BRAF, NRAS, NF1, JAK1, JAK2, PTEN, IFNGR1, IFNGR2 and IRF1, in skin melanoma.
