# Supplementary material for: Genomic landscape of the immunogenicity regulation in skin melanomas with diverse tumor mutation burden
Source: Front Immunol. 2022 Oct 28;13:1006665. doi: 10.3389/fimmu.2022.1006665 (PMC9650672; doi:10.3389/fimmu.2022.1006665)

primary melanoma
(top 250 downregulated genes)

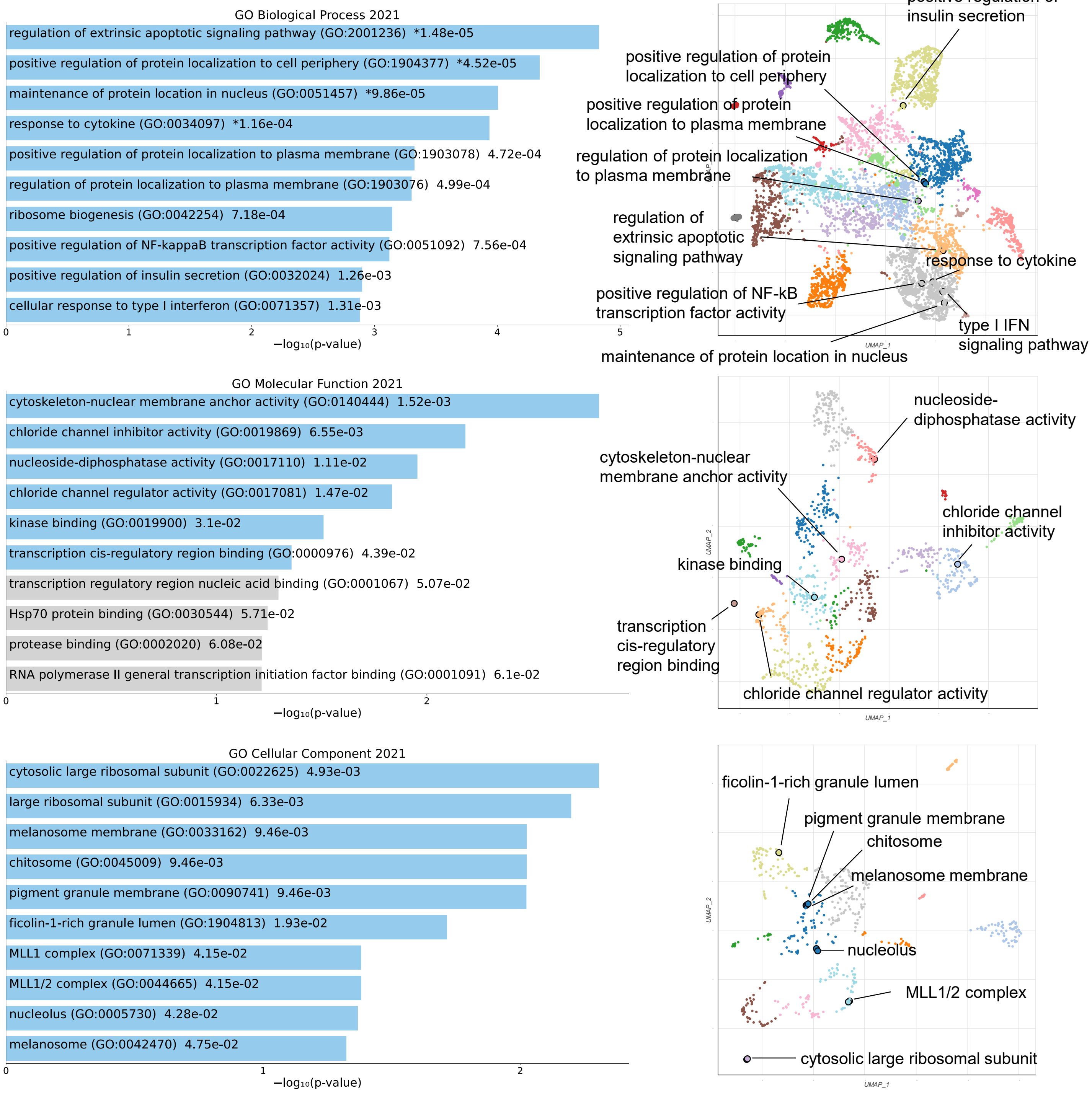

Supplement: Supplementary Figure 2 — The bar charts (left) depict the top 10 enriched Gene Ontology (GO) terms in the top 250 downregulated genes in primary skin melanoma, along with their corresponding p-values. Colored bars correspond to terms with significant p-values (<0.05). Asterisks (*) indicate the terms with significant adjusted p-values (<0.05). The scatterplots (right) were created using UMAP and are organized so that similar gene sets are clustered together. Larger, black-outlined points represent significantly enriched terms, the associated gene set names and p-values of which, are denoted. [file Image_2.pdf]
