# Supplementary figures and images for: Genomic landscape of the immunogenicity regulation in skin melanomas with diverse tumor mutation burden
Source: Front Immunol. 2022 Oct 28;13:1006665. doi: 10.3389/fimmu.2022.1006665 (PMC9650672; doi:10.3389/fimmu.2022.1006665)

metastatic melanoma  
(top 250 upregulated genes)

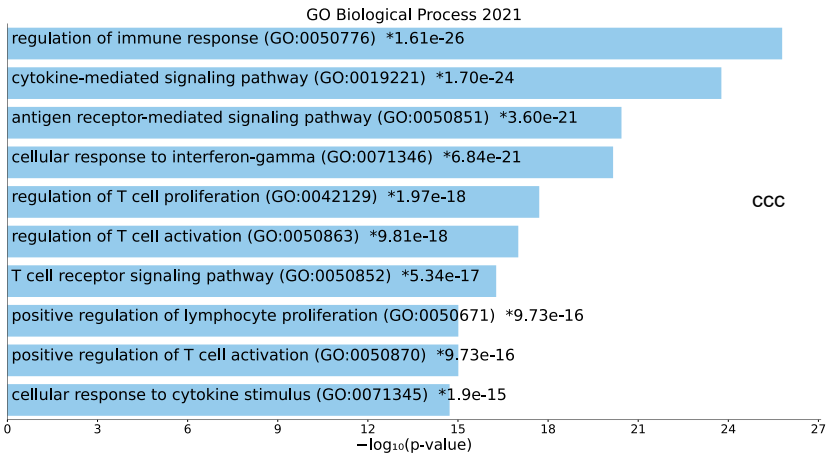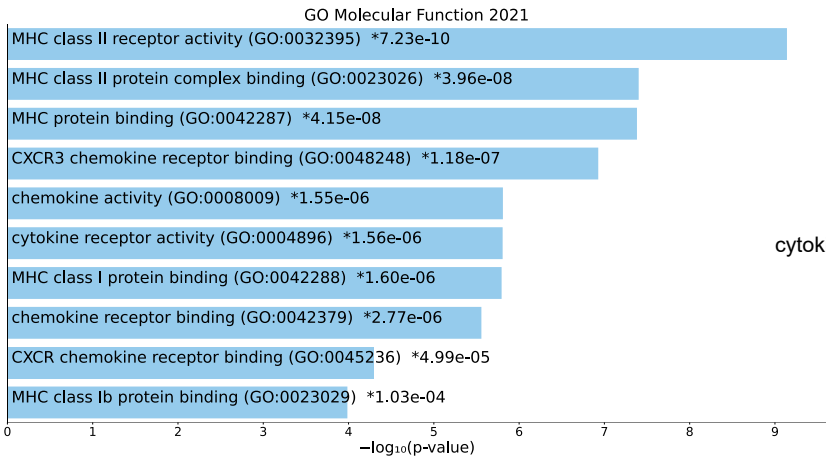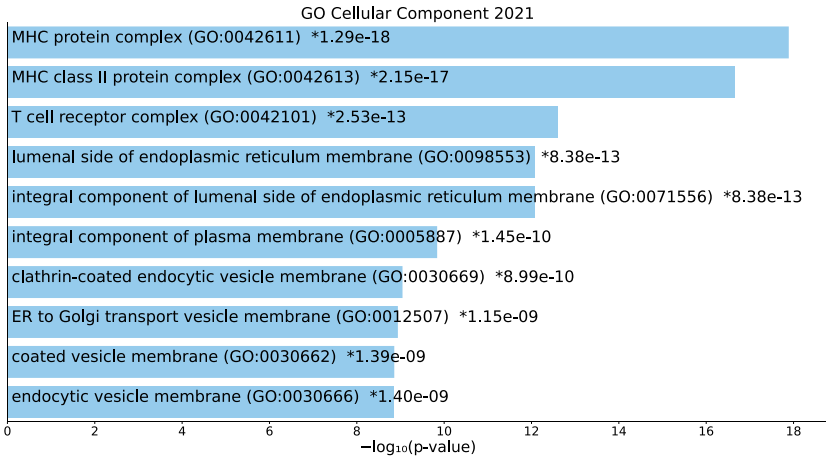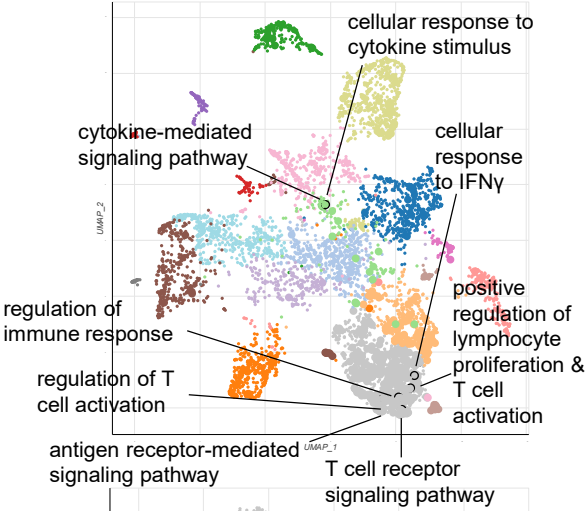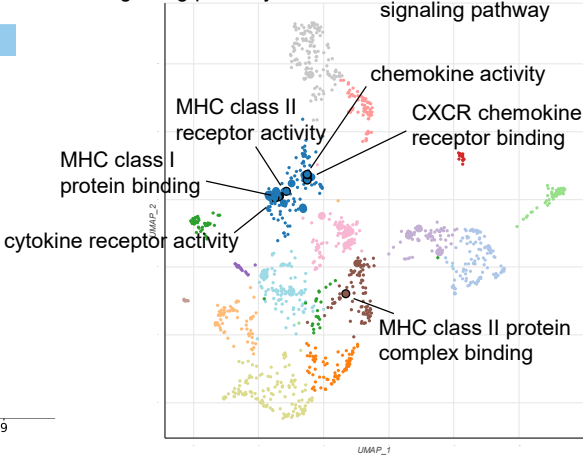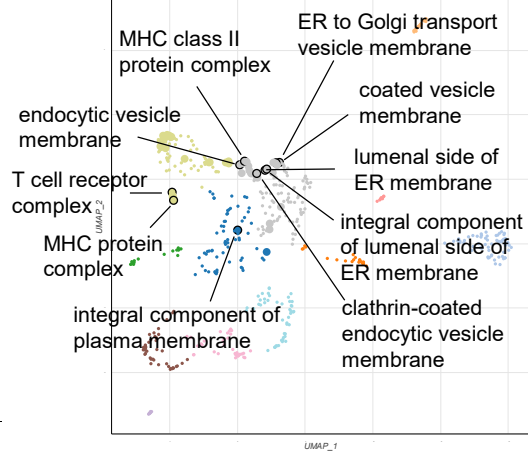

Supplement: Supplementary Figure 3 — The bar charts (left) depict the top 10 enriched Gene Ontology (GO) terms in the top 250 upregulated genes in metastatic skin melanoma, along with their corresponding p-values. Colored bars correspond to terms with significant p-values (<0.05). Asterisks (*) indicate the terms with significant adjusted p-values (<0.05). The scatterplots (right) were created using UMAP and are organized so that similar gene sets are clustered together. Larger, black-outlined points represent significantly enriched terms, the associated gene set names and p-values of which, are denoted. [file Image_3.pdf]

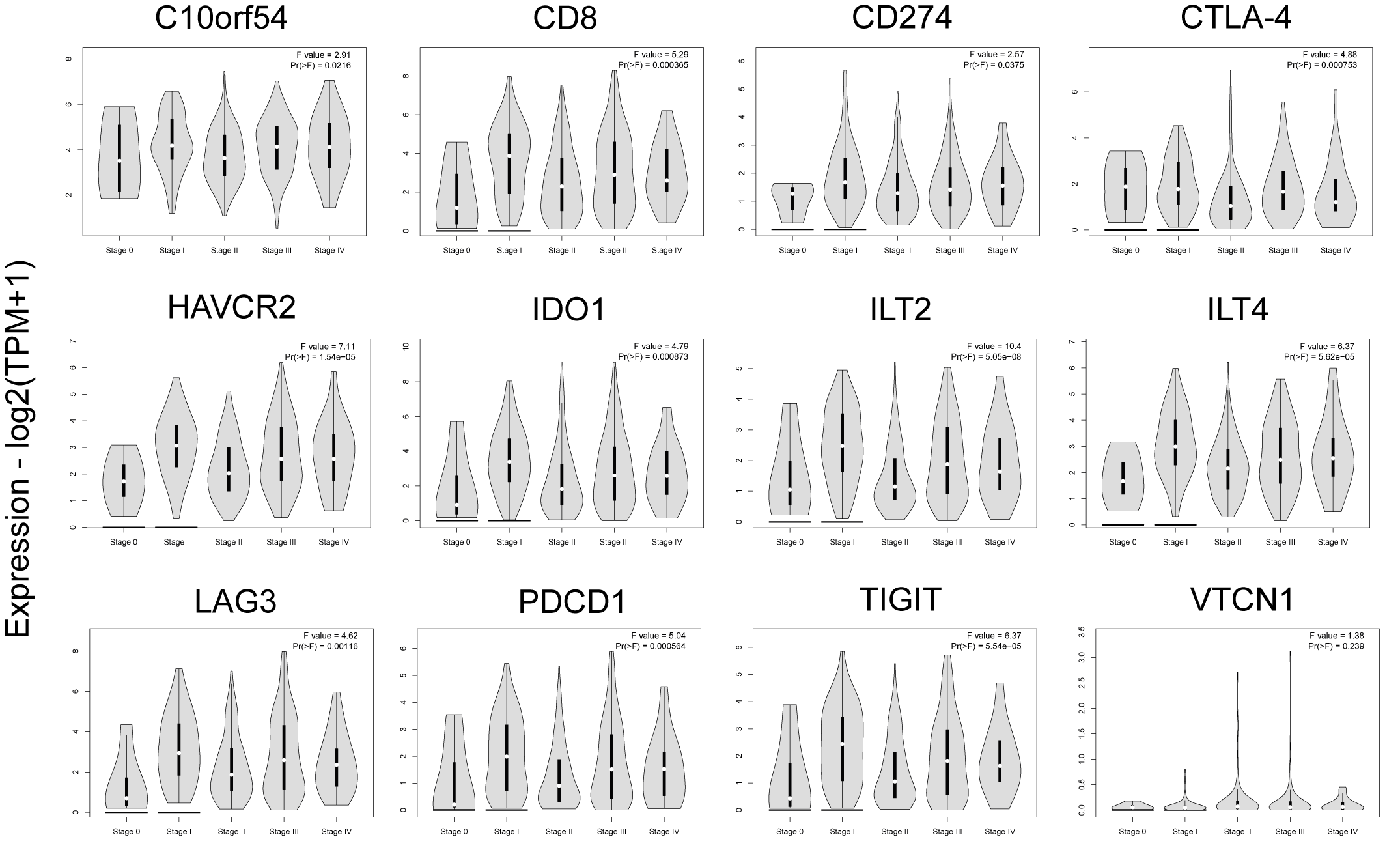

Supplement: Supplementary Figure 5 — Stage-plot analysis of the expression of CD8 and several immune receptors in skin melanoma, showing no significant differences according to the tumor’s stage. [file Image_5.tif]

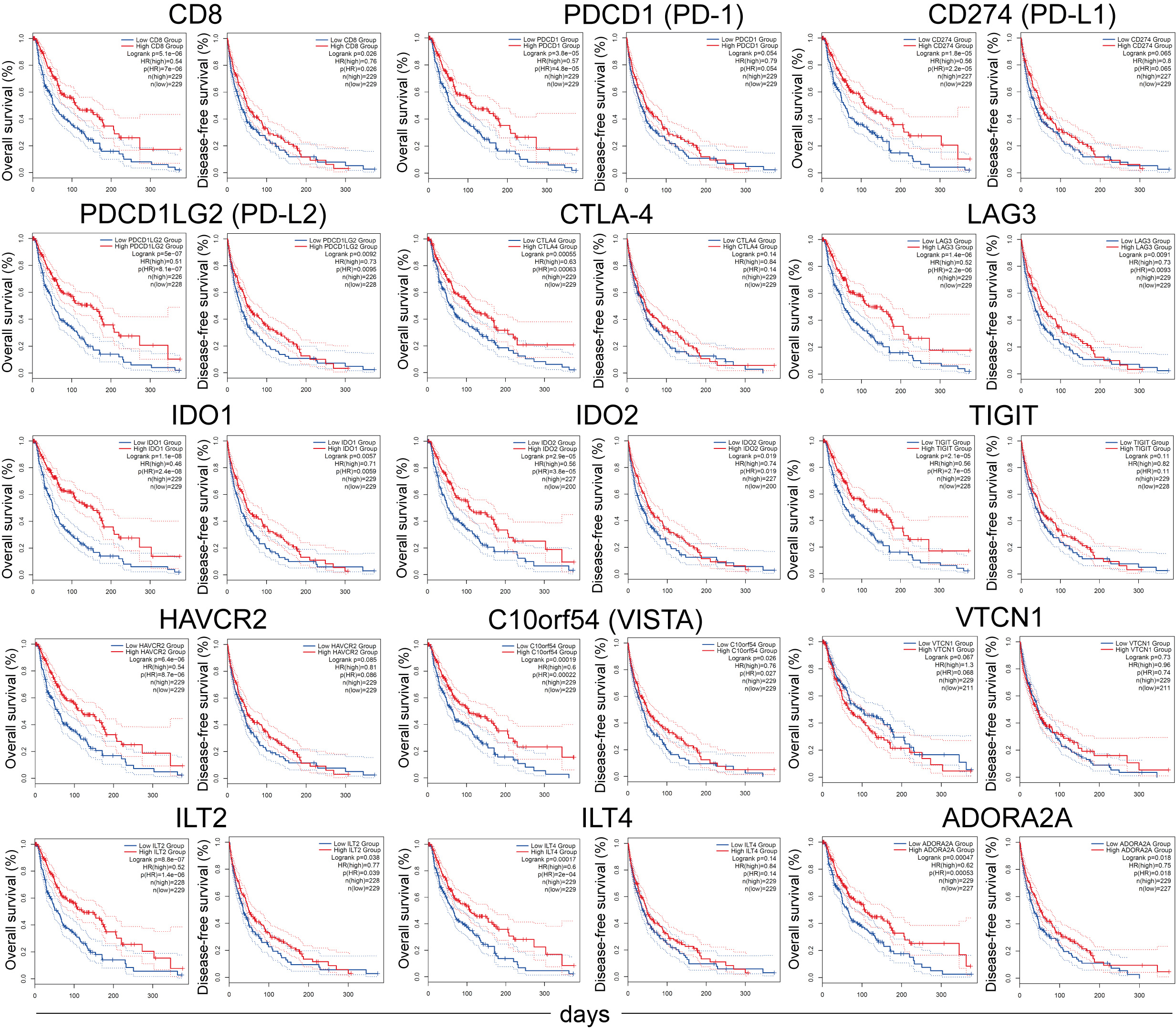

Supplement: Supplementary Figure 6 — The Kaplan-Meier curves show the overall and disease-free (DF) survival of melanoma patients, expressing high or low expression levels of PD-1, PD-L1/L2, CTLA-4, LAG3, IDO1/2, TIGIT, HAVCR2, VISTA, VTCN1, ILT2/4, ADORA2A and CD8 (marker for CD8+ T cells). The log-rank test was used to assess statistical differences between the two subgroups of patients. The patients were separated into high expression group (upper 50 percentile, red curve) and low expression group (lower 50 percentile, blue curve) by gene expression levels. The numbers of the patients in each group are provided as “n(high)” and “n(low)”, respectively. The log-rank p-value, along with the HR(high) and p(HR) values are also provided in each Kaplan-Meier survival plot. A Bonferroni-corrected cut-off log-rank p-value of <0.05 indicates statistical significance. [file Image_6.jpeg]

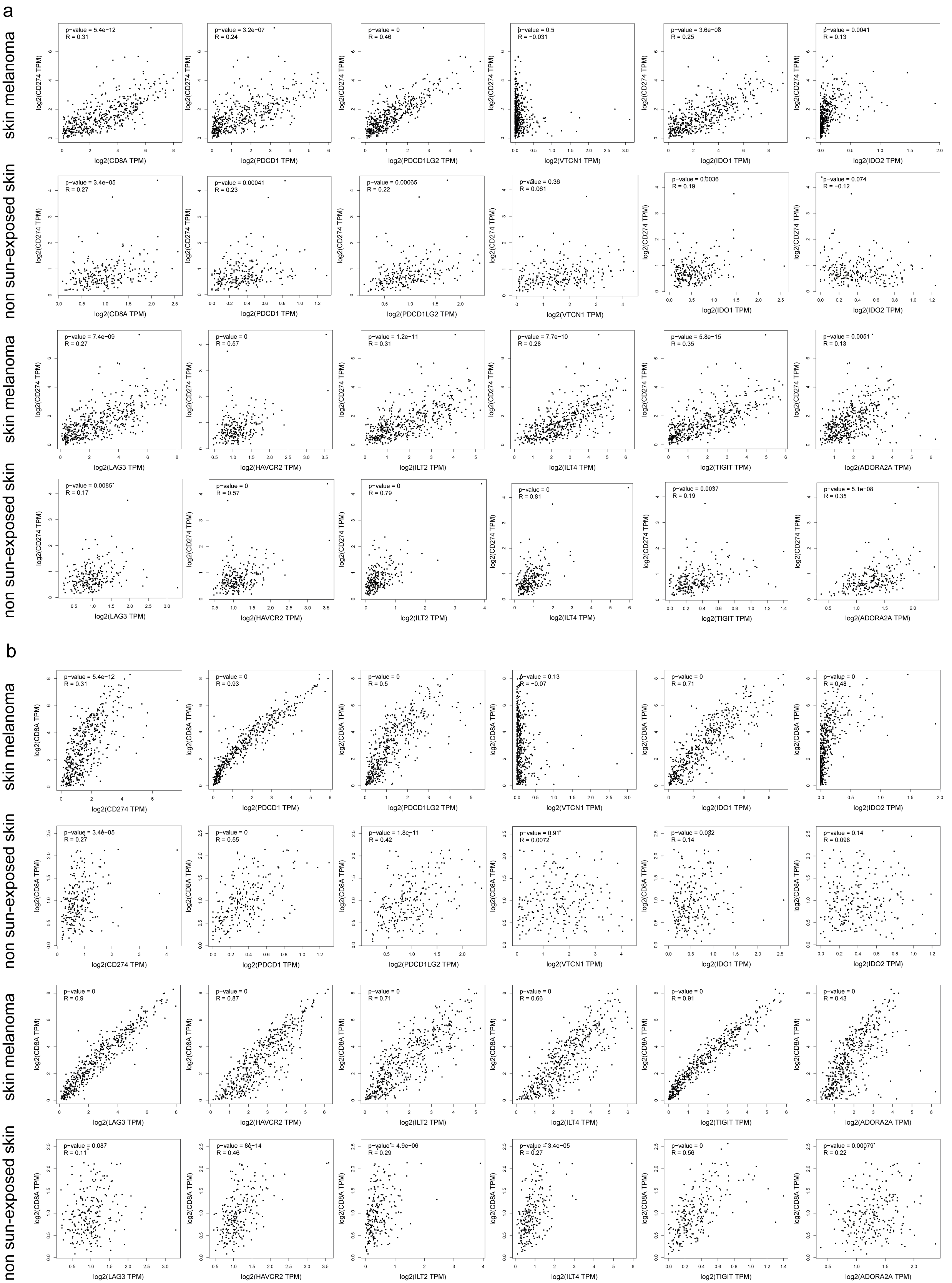

Supplement: Supplementary Figure 7 — The scatterplots depict the Pearson’s correlation coefficient (R and p-values) between the expression of CD274 (PD-L1) (A) or CD8A (B) and various immune receptors in skin melanomas (TCGA-SKCM) and normal suprapubic skin sample, not exposed to the sun (GTEx). [file Image_7.tif]

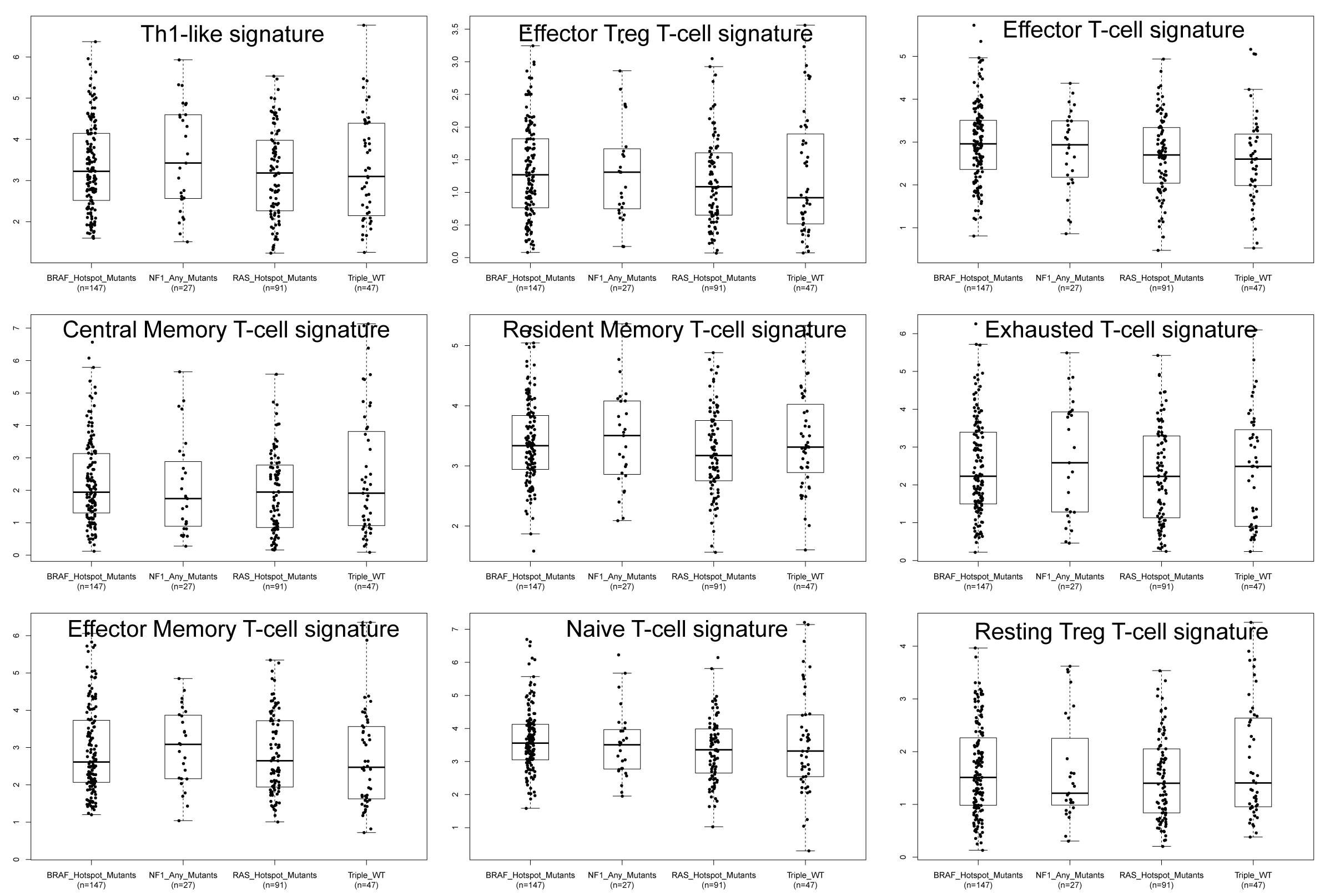

Supplement: Supplementary Figure 8 — The boxplots depict nine immune-signatures which did not differ across BRAFmut, NF1mut, RASmut and TripleWT skin melanoma tumors. Signatures were calculated in log2(TPM+1) using the |log2FC>1| and p<0.01 (ANOVA) as thresholds for statistical significance across the different skin melanoma subtypes. [file Image_8.tif]

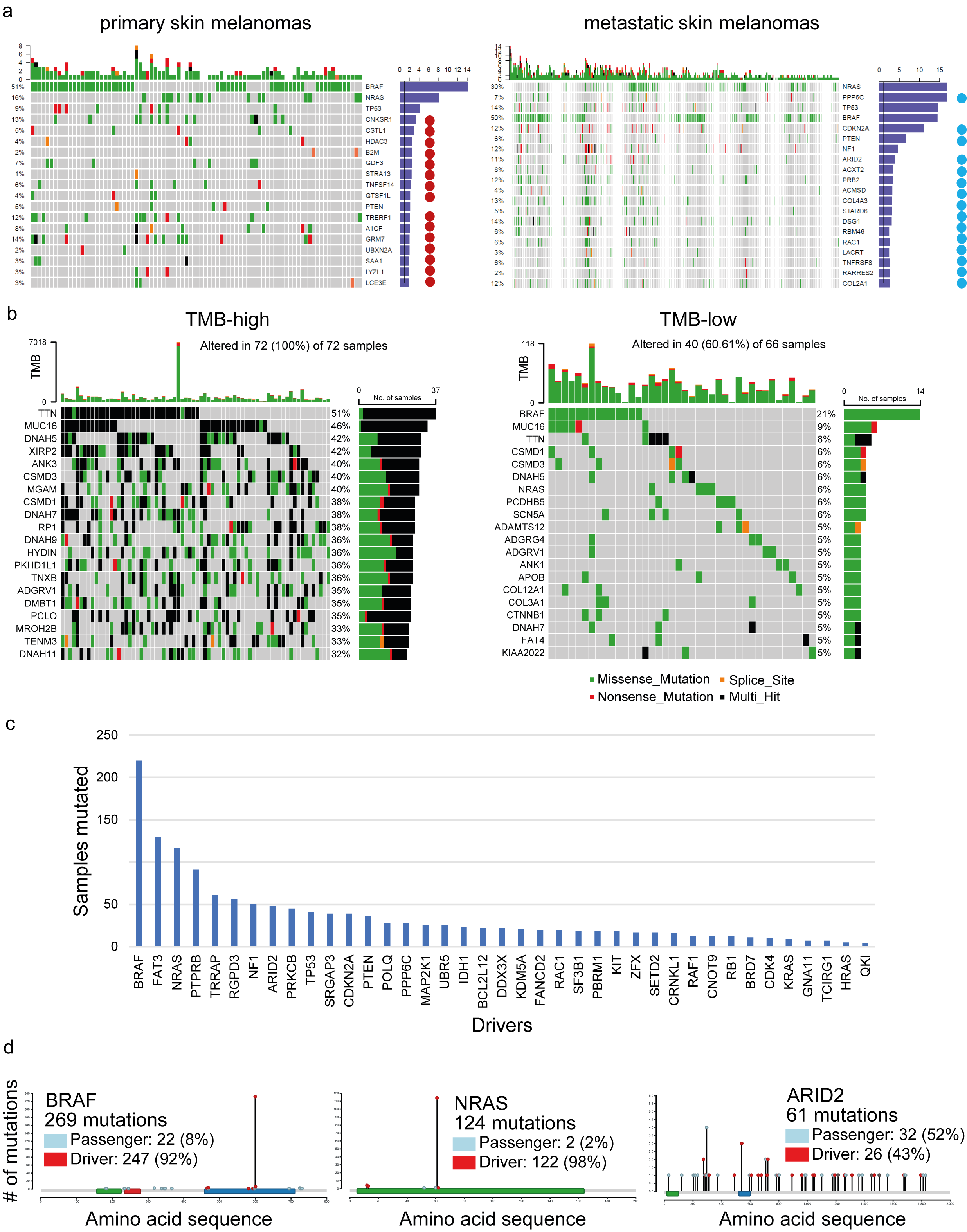

Supplement: Supplementary Figure 9 — The comutation plots depict the top 20 significantly mutated genes (SMGs, FDR<0.1) in primary and metastatic (A) or TMBhigh and TBlow skin melanomas (B). Green, red, pink, black and orange boxes indicate missense, nonsense, translation start site, multi-hit and splice-site mutations, respectively. The SMGs that correlate with primary or metastatic tumors (p<0.05) are highlighted by red or blue circles, respectively, next to the gene names. Each SMG’s q-values (−log10(FDR)) are plotted as a right-side bar plot in blue color. (C) The bar chart depicts the top 30 cancer drivers in skin melanoma. (D) The lollipop plots (below) report all the variants affecting the coding region of three drivers in skin melanoma (BRAF, NRAS and ARID2). Diagram circles are colored with respect to the corresponding mutations. Passenger mutations are highlighted in light blue and ad driver mutations in red. [file Image_9.tif]

# Immunostimulators

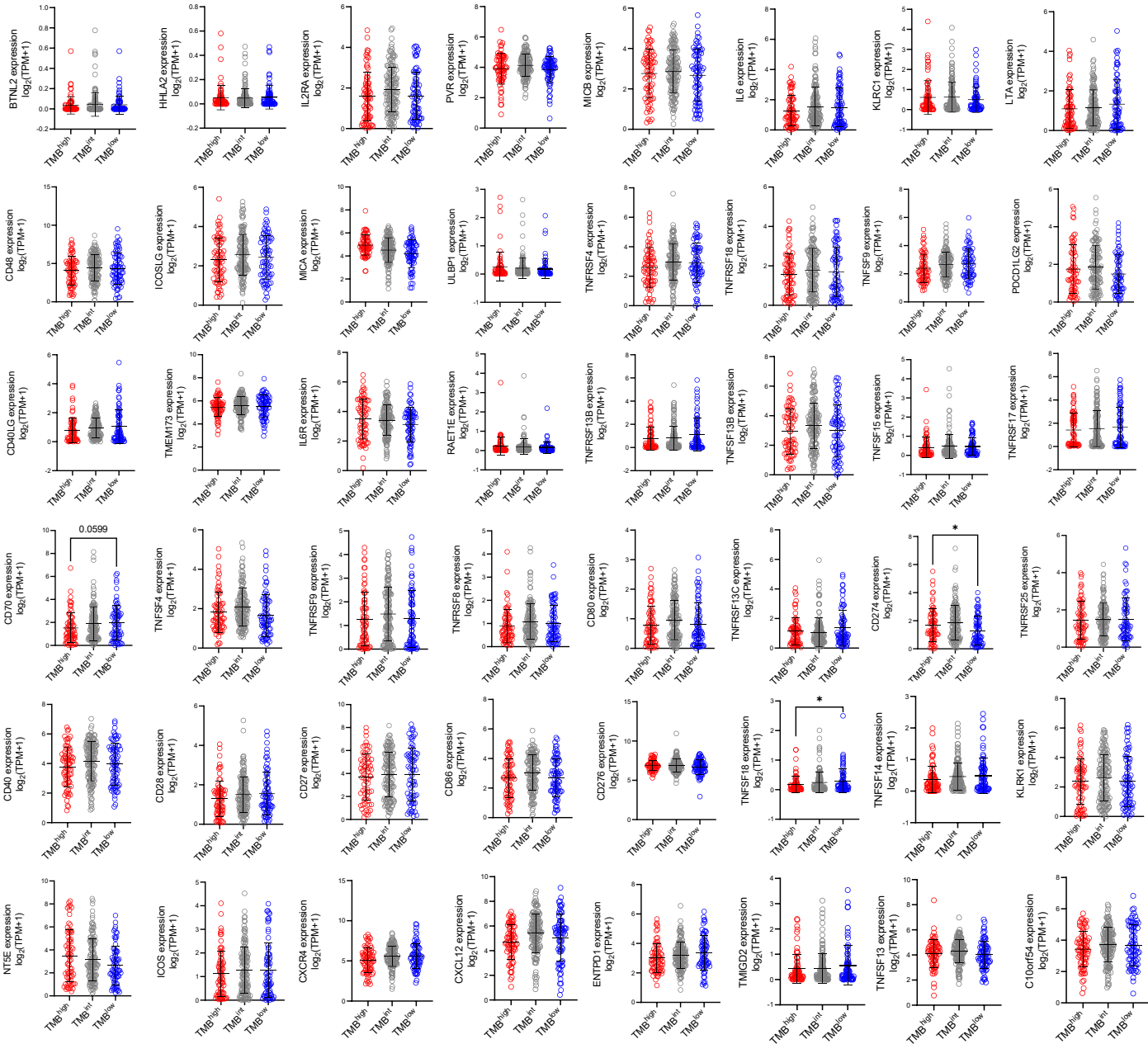

Supplement: Supplementary Figure 10 — The scatterplots show mean values in log2(TPM+1) with standard deviation (SD) of gene expression across various immunostimulators. Gene expression did not change across TMBhigh, TMBint and TMBlow skin melanomas, apart from CD274 and TNFSF18 (*, p<0.05. **, p<0.01). [file Image_10.pdf]

# Immunoinhibitors

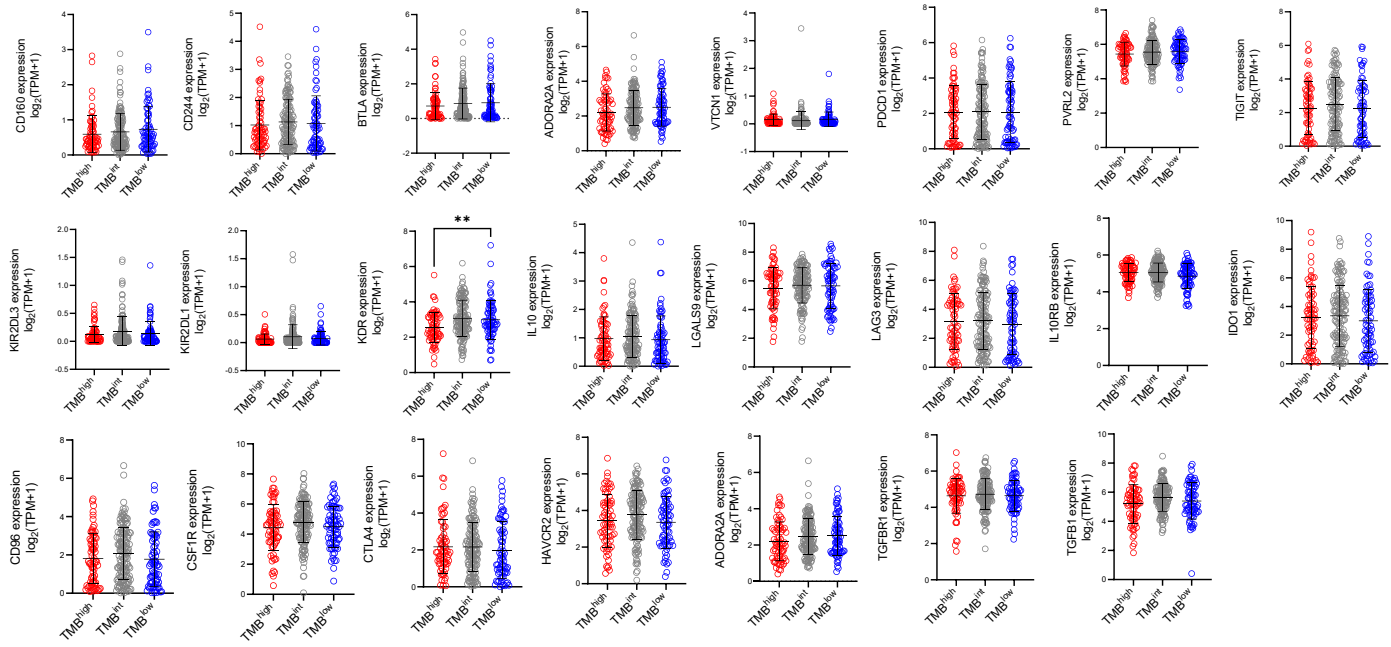

Supplement: Supplementary Figure 11 — The scatterplots show mean values in log2(TPM+1) with standard deviation (SD) of gene expression across various immunoinhibitors. Gene expression did not change across TMBhigh, TMBint and TMBlow skin melanomas, apart from KDR, which was lower in TMBhigh tumors (**, p<0.01). [file Image_11.pdf]

# Immunostimulators

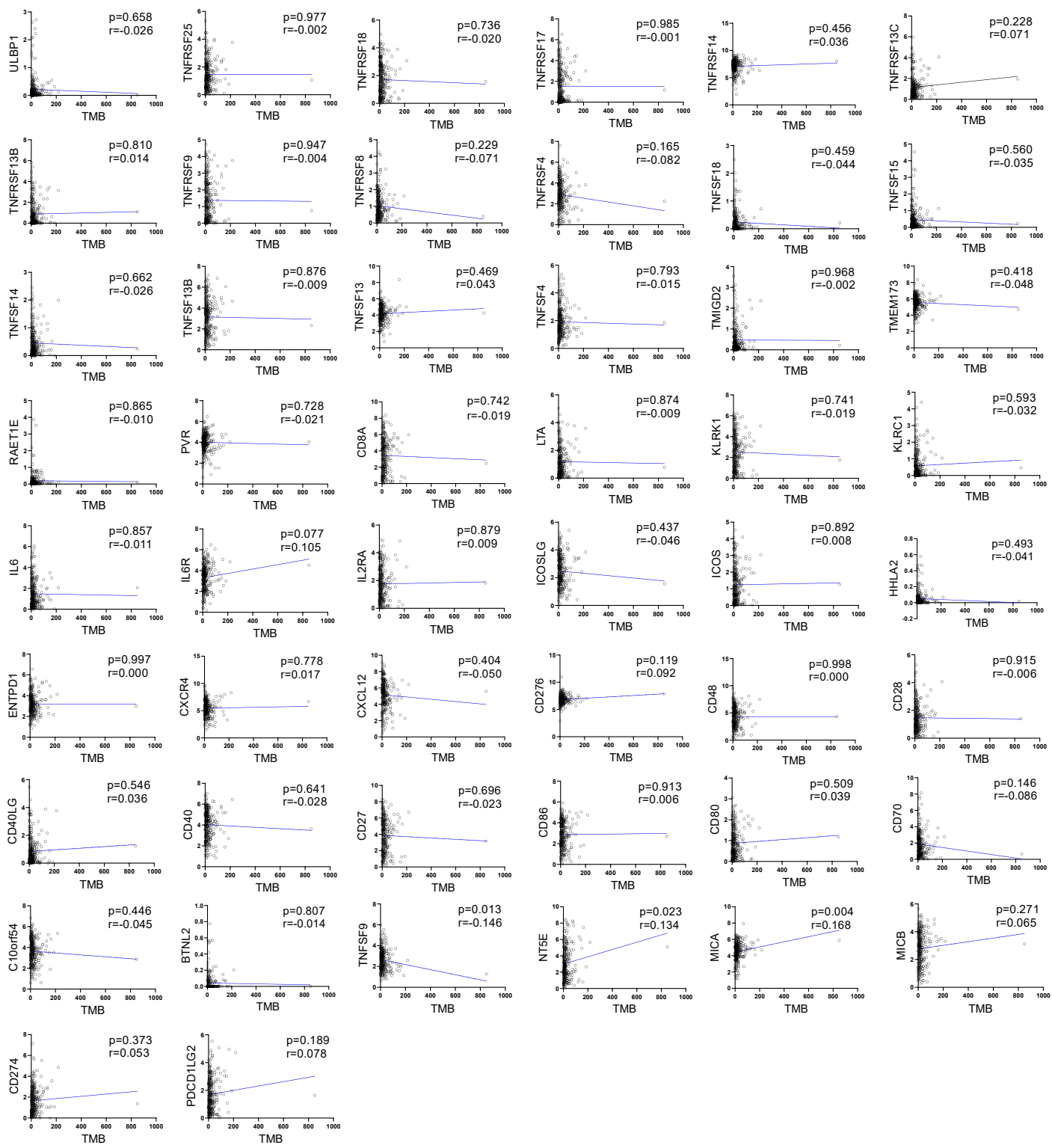

Supplement: Supplementary Figure 12 — Pearson’s correlation between TMB and the expression of activating immune receptors (immunostimulators), shows that there was no relationship between their gene expression and the TMB in skin melanoma. [file Image_12.pdf]

# Immunoinhibitors

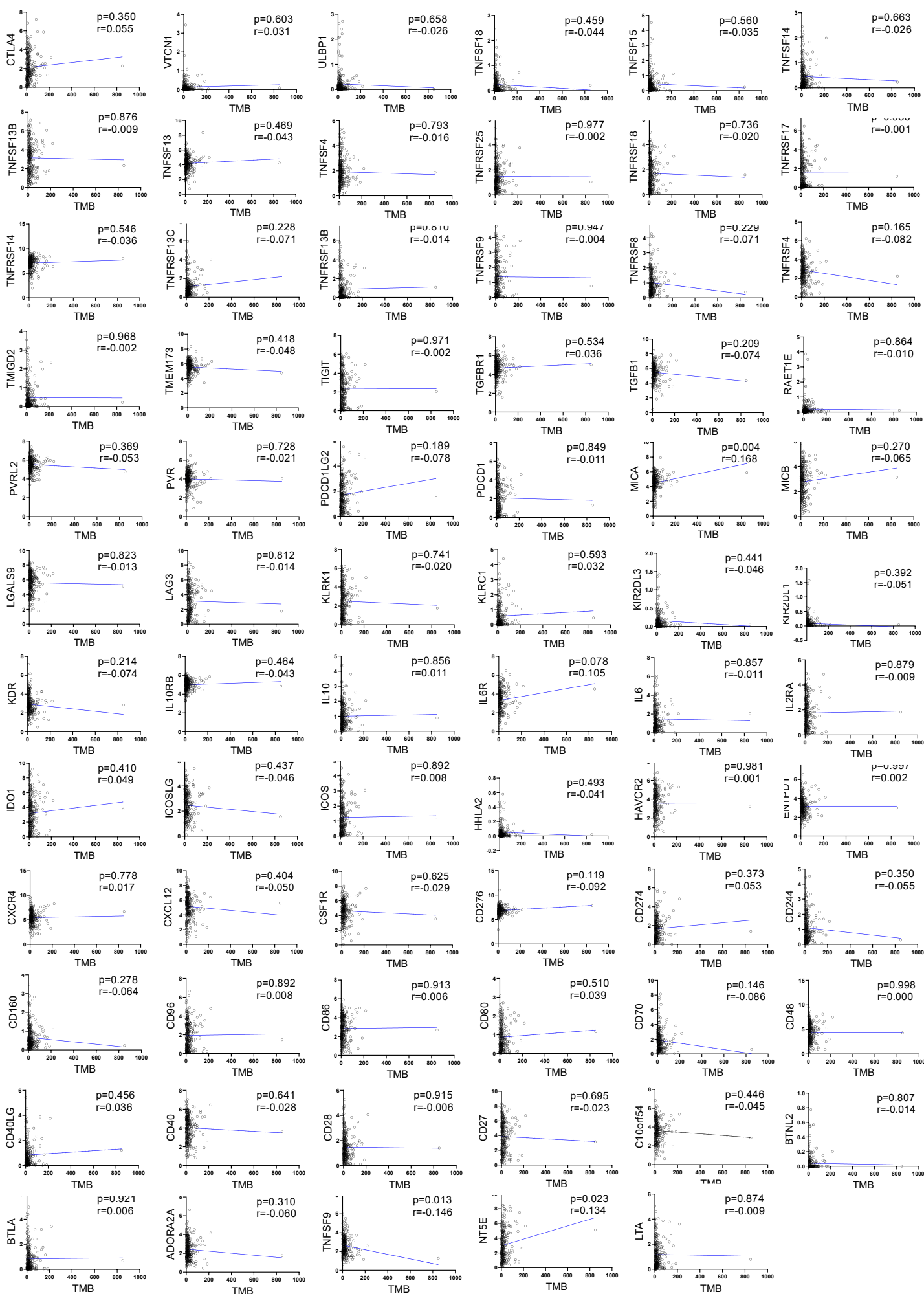

Supplement: Supplementary Figure 13 — Pearson’s correlation between TMB and the expression of inhibitory immune receptors (immunoinhibitors), shows that there was no relationship between the gene expression and the TMB in skin melanoma. [file Image_13.pdf]
